# Grad-CAM based deep learning analytics for image-level colon disease classification based on graph neural networks and vision transformers

**DOI:** 10.3389/fphys.2026.1734299

**Published:** 2026-05-25

**Authors:** Chaohui Zhen, Canhua Yao, Song Li, Zihong Lin, Umar Muhammad Ibrahim, Kavimbi Chipusu, Biao Zheng, Rui Liang

**Affiliations:** 1 Department of Gastrointestinal Surgery, The First Dongguan Affiliated Hospital, Guangdong Medical University, Dongguan, Guangdong, China; 2 Department of Surgery, The First Dongguan Affiliated Hospital, Guangdong Medical University, Dongguan, Guangdong, China; 3 School of Artificial Intelligence and Automation, Huazhong University of Science and Technology, Wuhan, China; 4 Department of Mechanical Engineering, Division of Biomedical Engineering, University of Saskatchewan, Saskatoon, SK, Canada

**Keywords:** colorectal cancer, endoscopy, Grad-CAM, graph neural networks (GNN), interpretability, Kvasir V2 dataset, medical image classification, vision transformer (ViT)

## Abstract

**Introduction:**

Accurate classification of colonoscopic images is essential for early detection and characterization of colorectal diseases. Recent advances in deep learning, particularly transformer-based architectures and graph neural networks (GNNs), provide alternative strategies for modeling global contextual information and relational structures in image representations. This study evaluates transformer-based and graph-based frameworks under a unified experimental protocol for endoscopic colon disease classification.

**Methods:**

Experiments were conducted on the Kvasir V2 dataset using two primary paradigms: (i) a Vision Transformer (ViT) with selective fine-tuning and learning-rate scheduling, and (ii) a CNN–GNN pipeline integrating image embeddings with graph construction strategies (cosine similarity, k-nearest neighbors, and epsilon-radius graphs) and multiple GNN architectures. Performance was evaluated using accuracy, precision, recall, and macro-F1 score, with Grad-CAM used for qualitative interpretability analysis.

**Results:**

The selectively fine-tuned Vision Transformer achieved 94.6% accuracy with a macro-F1 score of 0.94. The best graph-based configuration (ViT embeddings with epsilon graph and GIN aggregation) achieved 92% accuracy and 0.92 macro-F1 score.

**Discussion:**

Transformer-based contextual modeling provides strong discriminative capability for image-level colon disease classification, while graph-based relational modeling offers competitive performance when paired with high-quality embeddings.

## 1 Introduction

Despite advances in prevention and screening, colorectal cancer remains a major contributor to global cancer morbidity and mortality (Brenner et al., 2014). It ranks as the third most diagnosed malignancy and the second leading cause of cancer-related deaths globally. In 2021 alone, CRC accounted for more than 2.1 million new diagnoses and over 1 million deaths worldwide. The burden of the disease is particularly high in certain regions, with East Asia, Western Europe, and North America reporting the highest number of cases and fatalities. Alarmingly, epidemiological trends suggest that the incidence and mortality rates of CRC are increasing rapidly in low- and middle-income countries, where access to healthcare infrastructure and specialized oncology services remains limited. Projections indicate a 60% increase in the global incidence of CRC by 2030, alongside an estimated 60% and 71.5% rise in rectal and colon cancer-related deaths by 2035.

**Figure 1:**
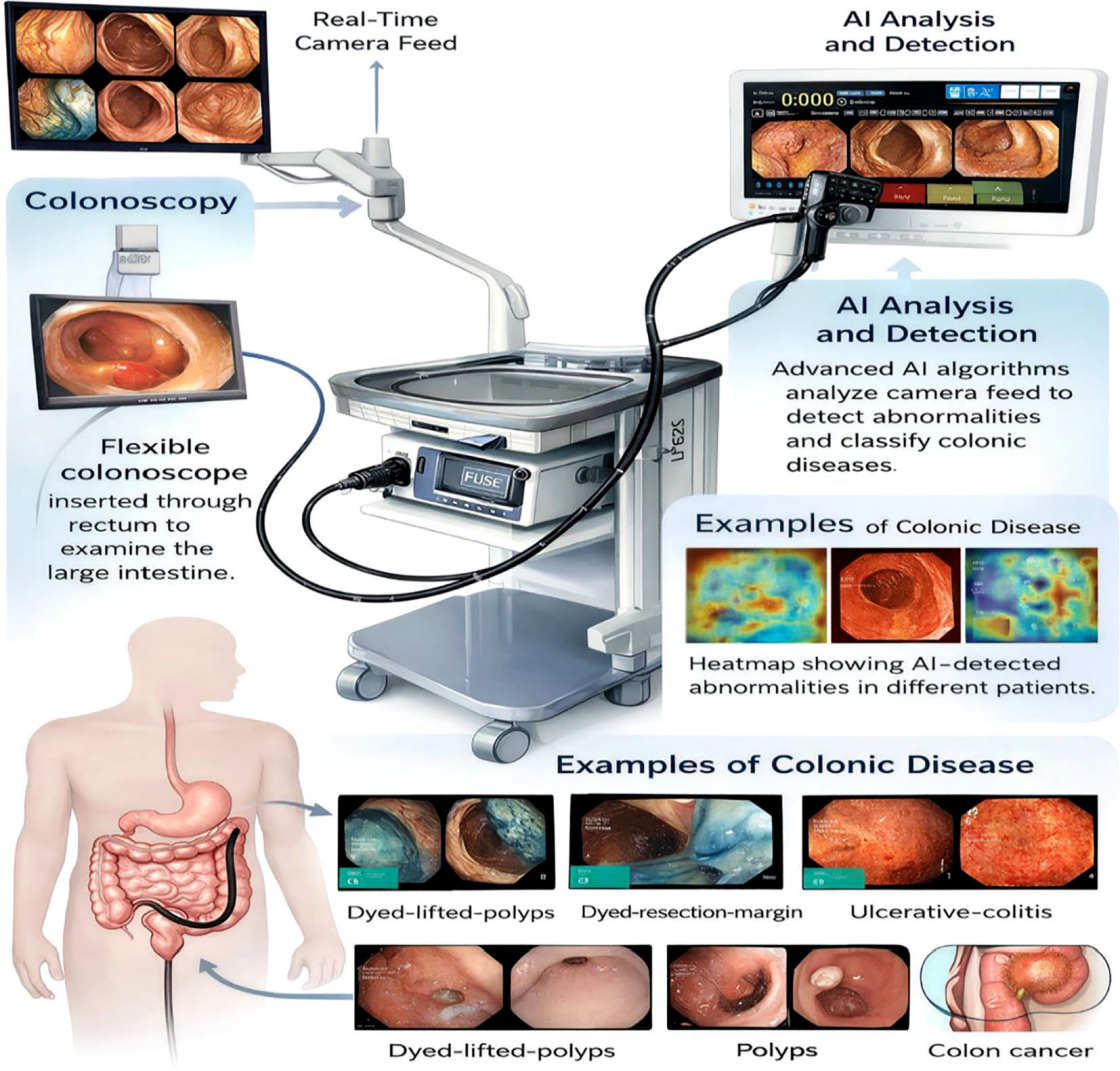
AI-driven colon disease classification using colonoscopy images. The workflow compares the AI-generated predictions of colon disease classifications with the ground truth labels. Grad-CAM visualizations display the regions of the colon images that influence the model’s prediction, such as polyp margins or areas of inflammation. The workflow ensures transparency and allows for an effective evaluation of model performance, aiding in the clinical decision-making process.

**Figure 1** illustrates the process of colonoscopy with integrated AI analysis. The flexible colonoscope, inserted through the rectum, captures real-time images of the large intestine. These images are processed by advanced AI algorithms to detect and classify colonic diseases, including dyed-lifted polyps, dyed-resection margins, ulcerative colitis, and polyps. Heatmaps highlight areas of detected abnormalities, enhancing diagnostic accuracy. The system combines traditional colonoscopy with AI-driven insights for improved disease detection and real-time analysis. The integration of deep learning in clinical settings is part of a broader shift toward intelligent healthcare systems and predictive modeling. Recent studies have demonstrated the efficacy of advanced mathematical modeling and computational intelligence in predicting disease trajectories and optimizing diagnostic workflows (Gandhi et al., 2025; Yan et al., 2025). These advancements highlight the necessity of robust frameworks that can handle complex medical data with high precision (Alzubaidi et al., 2025; Kumar et al., 2025a).

Research presents the mortality trends for colorectal cancer over a 20-year period, showing the breakdown of 153,490 total deaths across different colon regions: Right-sided colon: 41,100 deaths (26.8%), Left-sided colon: 38,100 deaths (24.8%), Rectosigmoid colon: 29,100 deaths (19.0%), and Unspecified colon: 45,190 deaths (29.4%) (Centers for Disease Control and Prevention (CDC), 2026). The chart also includes projections of age-standardized mortality rates, showing an increase of 60.1% by 2030 and 71.3% by 2035. These projected increases reflect the continued challenges in early detection and the growing global burden of CRC.

Early diagnosis is crucial as prognosis and survival rates are highly stage dependent. When identified at a localized stage, the 5-year survival rate exceeds 90%, whereas distant-stage colorectal cancer is associated with substantially poorer survival (National Cancer Institute, 2026). This surveillance role is especially important in ulcerative colitis, where the long-term risk of colorectal cancer is increased and careful endoscopic monitoring is clinically warranted (Jess et al., 2012). However, the accuracy and reliability of colonoscopy depend on factors such as the experience of the operator, the quality of bowel preparation, and the attention paid to subtle lesions (Wang et al., 2014). Additionally, colonoscopy is time-consuming, resource-intensive, and requires specialized training, which makes it less practical in resource-limited health systems. These challenges are even more pronounced in areas with a shortage of qualified gastroenterologists, leading to missed lesions, observer variability, and challenges in standardizing diagnostic quality issues that can have detrimental effects on patient outcomes. Early clinical experience has shown that artificial intelligence-assisted colonoscopy can support real-time polyp detection and reduce the likelihood of missed lesions (Misawa et al., 2018). These systems aim to reduce human errors, enhance diagnostic consistency, and lessen the workload of clinicians (Ozawa et al., 2020). Various studies have shown thepromising results of the models in different applications (Albuquerque et al., 2025; Benarba et al., 2015).

In recent years, deep learning (DL) techniques have shown significant promise in medical image analysis. DL models, particularly convolutional neural networks (CNNs), have demonstrated state-of-the-art performance in tasks such as image classification, object detection, and segmentation. Their ability to automatically learn hierarchical feature representations has made them highly effective in medical imaging applications. Furthermore, deep neural networks have achieved remarkable success in other fields such as natural language processing (Deng et al., 2009), speech and sound recognition, and interpretation of various biomedical imaging modalities, including MRI (Lundervold and Lundervold, 2019), CT scans (Würfl et al., 2016), histopathological slides (Mazaki et al., 2024), and gastrointestinal endoscopy (Min et al., 2019). The availability of large, publicly accessible datasets has facilitated the development of robust deep learning-based diagnostic systems for CRC. One of the widely used benchmarks for gastrointestinal image analysis is the Kvasir dataset (Pogorelov et al., 2017), which contains thousands of labeled images from routine clinical endoscopic examinations, covering categories such as polyps, stained and lifted polyps, cecum views, resection margins, and various anatomical landmarks. Other datasets, such as Kvasir-SEG (Jha et al., 2019) and LC25000 (Mazaki et al., 2024), have also contributed to the rapid development of automated detection systems by providing diverse, well-annotated training data. These datasets are essential for enabling reproducible research and for cross-algorithm performance comparisons.

Despite the significant advancements with CNN-based models, limitations still exist in effectively capturing relational dependencies among images and enhancing global/contextual visual understanding. To address these challenges, this paper introduces a novel colon disease classification framework that integrates CNNs with graph neural networks (GNNs). CNNs are used for feature-rich visual feature extraction from endoscopic images, while GNNs model the relationships between images through various graph construction methods, including cosine similarity, ϵ-radius graphs, and *k*-nearest neighbors. Relational modeling through GNNs helps capture similarities and differences among patient samples, which is particularly useful for distinguishing fine-scale pathological patterns. Additionally, this framework incorporates the use of Vision Transformers (ViT), which have shown superior performance in capturing long-range dependencies and global context within an image. ViT’s ability to aggregate visual features across the entire image field offers an improved approach to visual classification tasks without requiring additional textual or cross-modal inputs. The latest improvements in model efficiency were demonstrated in the works of Xu et al. (2020) and Zhang et al. (2025).

To provide interpretability for the system’s decision-making process, we apply Gradient-weighted Class Activation Mapping (Grad-CAM) to generate visual explanations of model predictions (Selvaraju et al., 2017). These saliency maps highlight the regions of the endoscopic images that most influence classification outcomes, allowing clinicians to verify that the model is focusing on clinically relevant structures. Such visualizations may assist in understanding model attention patterns, although formal validation through expert annotation or robustness testing would be required to establish clinical utility. These models have demonstrated robust performance across diverse use cases (Rozemberczki et al., 2019; Sawicki et al., 2021). In summary, this study presents a hybrid deep learning pipeline that combines CNNs, GNNs, and ViT-based visual feature aggregation for classifying colon diseases from endoscopic images in the Kvasir V2 dataset. Through the integration of powerful feature extraction, relational modeling, and visual learning, our proposed method aims to achieve high diagnostic accuracy while maintaining interpretability. This approach addresses key challenges in CRC detection and has the potential to serve as an invaluable decision-support tool for gastroenterologists, particularly in resource-limited settings where access to expert interpretation is constrained. Advancements in machine learning techniques have contributed to the enhancement of model performance (Kim et al., 2022; Kumar et al., 2025b).

Our contributions are as follows: This paper presents a comparative evaluation of CNN–GNN pipelines with varying graph construction methods (cosine similarity, ϵ-radius, and *k*-nearest neighbors) and multiple GNN architectures (GCN, GAT, GraphSAGE, and GIN). This paper explores ViT–Transformer-based visual classification models (a baseline ViT–Transformer and a tuned variant) for colon disease classification. Additionally, a benchmark evaluation of all models is conducted using precision, recall, F1-score, and accuracy, along with qualitative analysis using confusion matrices and Grad-CAM heatmaps.

## 2 Related work

Recent advancements in deep learning have brought about transformative changes in the classification and segmentation of colon diseases, especially colorectal cancer (CRC) and polyps. Among these innovations, Convolutional Neural Networks (CNNs), transformer-based models, and transfer learning techniques have gained significant attention. These methods offer enhanced diagnostic precision, reduced processing time, and increased resilience across various imaging conditions. They not only improve workflows in highly developed healthcare systems but also show potential for use in resource-limited settings, where access to specialized medical expertise is scarce. The integration of these technologies has helped reduce the heavy diagnostic workload on clinicians, providing results that are more consistent than traditional manual interpretation of medical images.

In this section, we examine recent developments in CNN-based models, transfer learning applications, and hybrid segmentation frameworks, highlighting both the progress achieved and the challenges that justify the current study. The evolution from initial CNN-based classification models to modern transformer and graph-based solutions is explored, emphasizing the need for models that, in addition to high accuracy, provide interpretability and relational understanding.

CNNs have found broad application in both histopathological and endoscopic imaging for CRC detection, proving highly effective for automatic feature extraction and classification. For instance, a study by Karthikeyan et al (Karthikeyan et al., 2024). involved a VGG16-based CNN model enhanced with feature-ranking algorithms, achieving a 92% accuracy rate in digital pathology datasets. This shows that even basic CNN architectures can perform effectively when augmented with additional mechanisms like feature ranking. However, while these algorithms improve feature importance estimation, they still fall short of providing interpretable decision pathways for clinicians.

Turning to colonoscopy image classification, Sharma et al (Sharma et al., 2020). devised a two-stage model, first identifying polyp frames and then categorizing them as neoplastic or non-neoplastic. This approach focused computational resources on clinically relevant images. Their tests using CNNs such as VGG16, InceptionV3, ResNet50, and DenseNet found VGG19 to be the most accurate. These findings reinforce the ability of CNNs to identify important features from complex images but also point out the limitation of relying solely on visual inputs, without incorporating other contextual information that could aid decision-making. CNNs in CRC detection have demonstrated some versatility, as evidenced by their application in breast cancer diagnosis. Alanazi et al (Alanazi et al., 2021). showed that a simple CNN model, trained on the Kaggle H2E dataset, achieved 87% accuracy, outpacing several traditional classifiers. Despite its simplicity, the model could identify pathology-related features, although its performance remained limited without advanced feature synthesis techniques.

Similarly, in lung cancer detection, Hatuwal et al (Hatuwal and Thapa, 2020). used CNNs to classify images from the LC25000 dataset, achieving a validation accuracy of 97.2%. While these results demonstrate the versatility of CNNs across various medical imaging tasks, they also reveal a common weakness: CNNs process each image individually, lacking the ability to recognize the relationships between different regions of tissue, which could be crucial in clinical contexts. The inherent “black box” nature of CNNs also makes it difficult for clinicians to fully trust their decisions, as the reasoning behind predictions is not transparent.

While Transformers and Graph-based models have seen success in medical imaging, their utility extends to complex sequence modeling and relational data analysis in other high-precision engineering fields. For instance, enhanced attention-based Transformers and temporal convolution models have been utilized for advanced prediction tasks, showcasing the versatility of these architectures in capturing long-range dependencies (Wang et al., 2025). Similarly, hybrid architectures combining variational autoencoders with Graph Attention Networks (GAT) have proven effective in extracting features from high-dimensional signal data (Shen et al., 2026). While standard CNNs excel at local feature extraction, current research emphasizes the need for capturing long-range spatial dependencies. This trend is consistent with transformer-based medical imaging frameworks such as UNETR, which demonstrated that transformer encoders can effectively model global context in medical image analysis (Hatamizadeh et al., 2022). Similarly, hybrid architectures that incorporate Graph-based relational reasoning have proven effective in extracting discriminative features by modeling the relationship between different anatomical structures (Zhu and Chen, 2025; Patel and Zhang, 2026).

Related hybrid deep learning and machine learning approaches have also shown strong performance for lung and colon histopathology classification, further supporting the value of transfer learning in cancer image analysis (Ochoa-Ornelas et al., 2024). By leveraging pre-trained models from large natural image datasets, transfer learning allows these models to be fine-tuned on smaller medical datasets. Ochoa-Ornelas et al (Ochoa-Ornelas et al., 2025). demonstrated robust transfer-learning performance using EfficientNet-based architectures for lung and colon cancer detection on histopathological datasets, highlighting how pre-training on larger datasets can enhance performance in specialized tasks. Hasan et al (Le et al., 2025). contributed to this by developing a compact multi-scale CNN (LW-MS-CCN) with only 1.1 million parameters, which still achieved strong performance, showing that smaller models can also reach state-of-the-art levels when paired with the right feature extraction strategies. These smaller models are suitable for use in low-resource settings or on mobile diagnostic devices. Overall, CNN-based transfer learning models remain highly effective, but they still struggle to capture relational dependencies and typically do not integrate non-visual clinical context, which limits their diagnostic completeness.

Recent deep learning studies have continued to emphasize early gastrointestinal polyp identification and segmentation as a clinically important direction for endoscopic image analysis (Dhrari Hajsalem and Ben Ayed, 2025). Recent work has explored hybrid convolution–transformer segmentation strategies to improve localization and boundary delineation in colonoscopy images. For example, Wang et al (Wang et al., 2025b). report strong segmentation performance using deep learning-based models for gastrointestinal polyp segmentation. Additionally, DCATNet integrates transformer-style modules and attention mechanisms for improved segmentation accuracy across common benchmarks (Wang et al., 2025a). PraNet further demonstrates the effectiveness of structured attention design for polyp segmentation in endoscopic imagery (Fan et al., 2020). These trends highlight the growing interest in hybrid models that combine local feature extraction and global reasoning to improve polyp segmentation performance.

While most studies have focused on closed-set classification and segmentation, open-set recognition (OSR) has also been explored as a clinically relevant direction, enabling models to better handle unknown or out-of-distribution categories. Moazzami et al (Moazzami et al., 2025). investigated open-set recognition for endoscopic image classification on the Kvasir dataset, emphasizing the importance of unknown-class awareness in realistic clinical environments. Similarly, Siddiqui et al (Siddiqui et al., 2025). developed SNet, a custom CNN model for endoscopic image classification, achieving high performance on Kvasir benchmarks. Hybrid models, such as those proposed by Khazaee Fadafen et al (Khazaee Fadafen and Rezaee, 2023), which combine deep feature backbones with additional classification strategies, have also demonstrated strong performance in histopathology-based CRC classification. Paladini et al (Paladini et al., 2021). used ensemble learning with pretrained CNNs for colorectal tissue classification, further supporting the effectiveness of ensemble strategies in improving accuracy and stability.

Despite these advancements, several challenges remain. Many current studies rely on isolated visual data, which may limit the model’s ability to capture the broader contextual visual relationships present across diverse patient samples. Additionally, while attention maps and saliency techniques offer partial interpretability, they are not sufficient to provide comprehensive clinical explanations, hindering their adoption in high-risk medical applications where trust is essential.

More broadly, deep learning and transfer learning approaches have shown usefulness across multiple medical imaging diagnosis tasks, reinforcing their relevance for automated clinical decision support (Kassani et al., 2019). AI-driven frameworks are increasingly being used to support clinical decision-making, predictive diagnostics, and intelligent healthcare monitoring systems. For example, recent research has explored deep learning–based models for disease prediction and medical data analysis, demonstrating how machine learning algorithms can improve diagnostic accuracy and patient outcome prediction in healthcare environments (Misra and Roy, 2024; Gandhi et al., 2025). Similarly, deep learning frameworks have been applied to various medical imaging and diagnostic tasks, including disease classification and healthcare data analytics, highlighting the growing importance of AI-assisted medical diagnosis (Alzubaidi et al., 2025; Kumar et al., 2025a). Intelligent healthcare systems integrating machine learning techniques have also been proposed to support automated health monitoring and predictive modeling for early disease detection (Rahman et al., 2025; Zhang and Li, 2025). These studies illustrate the expanding role of artificial intelligence in healthcare diagnostics and motivate the development of robust and interpretable deep learning frameworks for medical image analysis.

From a methodological perspective, GNNs are particularly suitable for modeling higher-order dependencies through message passing over graph-structured data (Wu et al., 2021). By representing datasets as graphs, with nodes corresponding to image-level embeddings and edges capturing similarity structure, GNNs can encode higher-order contextual information that traditional CNNs or transformers may overlook. GNN-based methods have proven effective in medical disease prediction tasks (Parisot et al., 2018). Motivated by the limitations of current models, this study presents a CNN–GNN pipeline designed to extract visual features and model relational dependencies among image samples. In addition, a transformer-based visual feature aggregation model is explored to enhance interpretability and performance for colon disease classification. By addressing the limitations of traditional single-image and low-interpretability approaches, this research seeks to provide clinically viable AI solutions for diagnosing and treating colorectal diseases.

## 3 Methodology

This study aims to propose a hybrid learning framework that combines graph-based learning with transformer-based visual feature aggregation for classifying colon diseases from endoscopic images. To achieve this, an end-to-end pipeline is presented, leveraging both CNN–GNN architectures and ViT–Transformer frameworks. The Kvasir V2 dataset serves as the primary image source, covering various colonoscopy scenarios, and is processed through multiple deep learning models for feature extraction, classification, and interpretability assessment.

**Figure 2:**
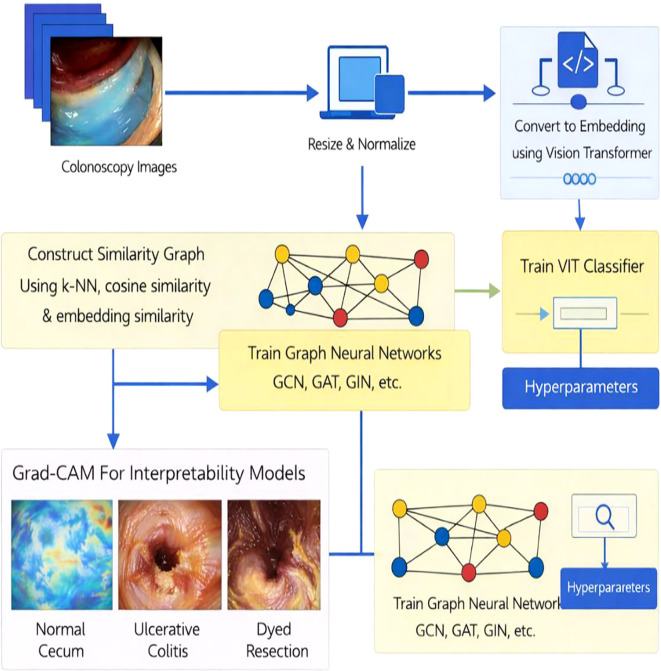
Architectural overview of the proposed framework for colon disease classification. The diagram illustrates the parallel processing of visual features through a Vision Transformer (ViT) and a Graph Neural Network (GNN) to achieve enhanced global and contextual visual understanding.

**Figure 2** illustrates the standard research workflow followed in this study. The pipeline is structured with two parallel branches. The first branch utilizes MobileNetV2 and ViT image feature embeddings, which are then used to build graphs through three methods: cosine similarity, ϵ-radius, and *k*-nearest neighbors. These graphs are subsequently passed through four different GNN variants GCN, GAT, GraphSAGE, and GIN for the final classification. The second branch focuses on transformer-based visual feature aggregation using ViT-based models, evaluating both base and fine-tuned versions of the ViT-Transformer model. The interpretability of both branches was examined using Grad-CAM, highlighting key regions in the endoscopic images. The overall process was assessed based on performance metrics such as accuracy, precision, recall, and F1-score.

### 3.1 Dataset description

**Figure 3:**
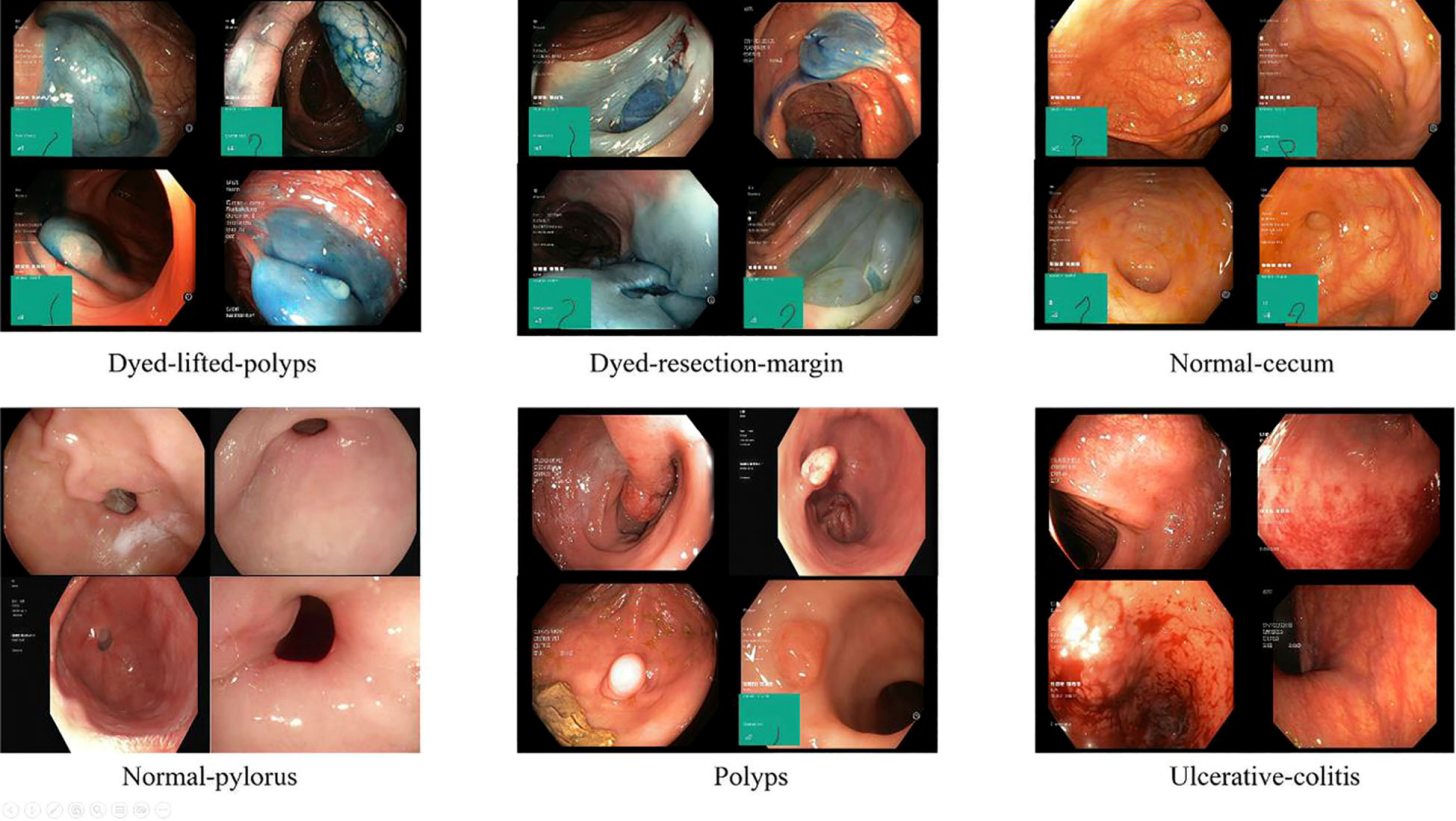
Sample colonoscopy images of all the classes from the dataset. The demonstration of various colonoscopy conditions: “dyed-lifted polyps,” where polyps are enhanced with dye and lifting techniques to assess morphology and resectability; “dyed-resection-margins,” which highlight the dyed margins of resected tissue to evaluate resection completeness; “polyp,” depicting standard polyps used for early detection and cancer prevention; and “ulcerative-colitis,” showing characteristic images of inflammation and ulceration, providing insights into disease activity in chronic bowel conditions.

For this study, the Kvasir V2 dataset, a publicly available collection of high-quality gastrointestinal (GI) endoscopic images, was used. This dataset contains images from real colonoscopy procedures and is extensively employed in medical image analysis for detecting, classifying, and segmenting diseases. It includes labeled examples of several diagnostic categories related to colon health. In this research, six distinct classes were chosen, with 1,000 images per class, totaling 6,000 images. **Figure 3** presents representative examples from each of these classes.

The dataset features a range of different endoscopic image classes, each serving a particular diagnostic function. The “dyed-lifted polyps” class includes images of polyps enhanced using dye and lifting techniques, helping to assess their morphology and resectability. The “dyed-resection-margins” class highlights the dyed margins of resected tissue, aiding in the evaluation of resection completeness. Anatomical reference classes, such as “normal-pylorus” and “normal-cecum,” showcase healthy anatomical structures to help models recognize normal features and reduce the occurrence of false positives. The “polyps” class contains images of standard polyps used for early detection and cancer prevention, while the “ulcerative-colitis” category features images of inflammation and ulceration, providing insights into the disease activity of chronic bowel conditions.

All images in the dataset are labeled with their corresponding class and stored in JPEG format with varying resolutions. The images were resized to a uniform input size for model training, and generalizability was improved using data augmentation techniques. The dataset was divided into training, validation, and test sets in an 80:10:10 ratio to enable robust evaluation.

### 3.2 Data preprocessing

**Figure 4:**
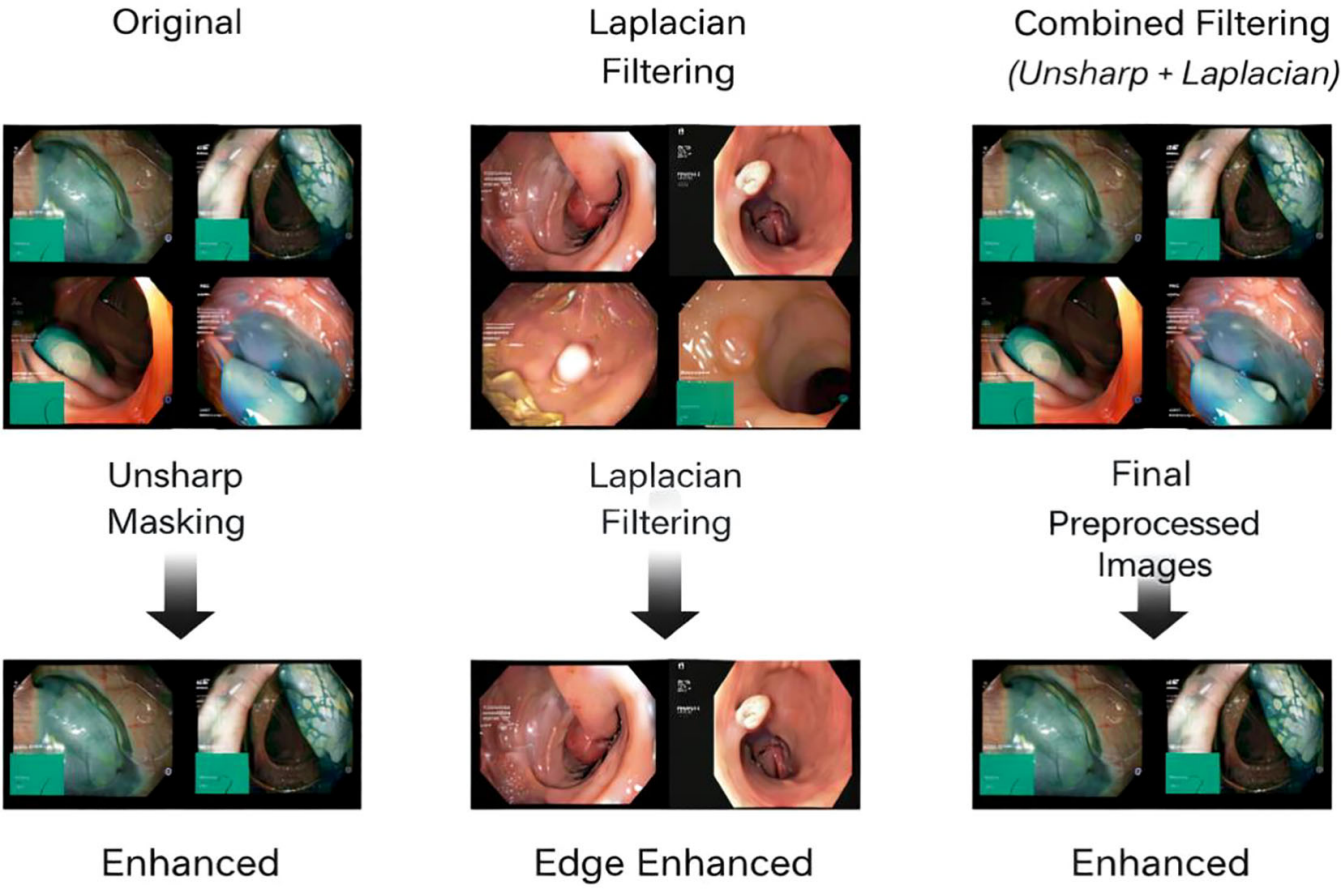
Preprocessing operations applied to endoscopic images using Unsharp Masking and Laplacian filtering. The top row shows representative original colonoscopy images from the dataset. The arrows indicate preprocessing operators applied to enhance structural features. The middle column illustrates the effect of Laplacian filtering, which highlights high-frequency edge components. The left column shows Unsharp Masking results that sharpen boundaries and improve contrast of subtle mucosal structures. The right column presents the combined enhancement output, where both operators are applied to improve visibility of disease patterns prior to feature extraction.

Preprocessing techniques were employed to improve the quality and clarity of input images before training the model. Two filtering methods, Unsharp Filtering and Laplacian Filtering were used to enhance the visibility of disease patterns in the endoscopic images. Unsharp Filtering was applied to sharpen the images by emphasizing edges and fine details, which helped to better distinguish between healthy and affected areas. Laplacian Filtering was used to highlight high-frequency components and enhance the image edges, making subtle texture variations more noticeable. The processed images generated by these techniques were used to form the refined dataset for subsequent analysis. **Figure 4** provides an example of the original image alongside the enhanced versions created using both filtering methods.

The preprocessing techniques applied in this study include unsharp masking and Laplacian filtering, both of which enhance the details of endoscopic images. These methods are widely used in medical imaging for edge detection and noise reduction. These preprocessing steps aim to enhance edge contrast and highlight fine structural patterns. However, their impact on model performance was empirically evaluated through an ablation study to ensure that improvements were not driven by artificial artifacts (Wang et al., 2014). These preprocessing steps are specifically designed to refine the image quality, thereby facilitating better feature extraction for subsequent classification and segmentation tasks.

#### 3.2.1 Preprocessing ablation study

To assess whether edge-enhancement techniques introduce artificial performance gains or unintended artifacts, we conducted an ablation study comparing model performance with and without unsharp masking and Laplacian filtering.

In the ablation setting, all training procedures, data splits, hyperparameters, and model architectures were kept identical. The only difference was the removal of the preprocessing filters, with images resized and normalized directly before training. This controlled comparison allows evaluation of whether performance improvements stem from genuine feature enhancement or from shortcut learning introduced by artificial edge amplification.

### 3.3 Description of the CNN-GNN model

**Figure 5:**
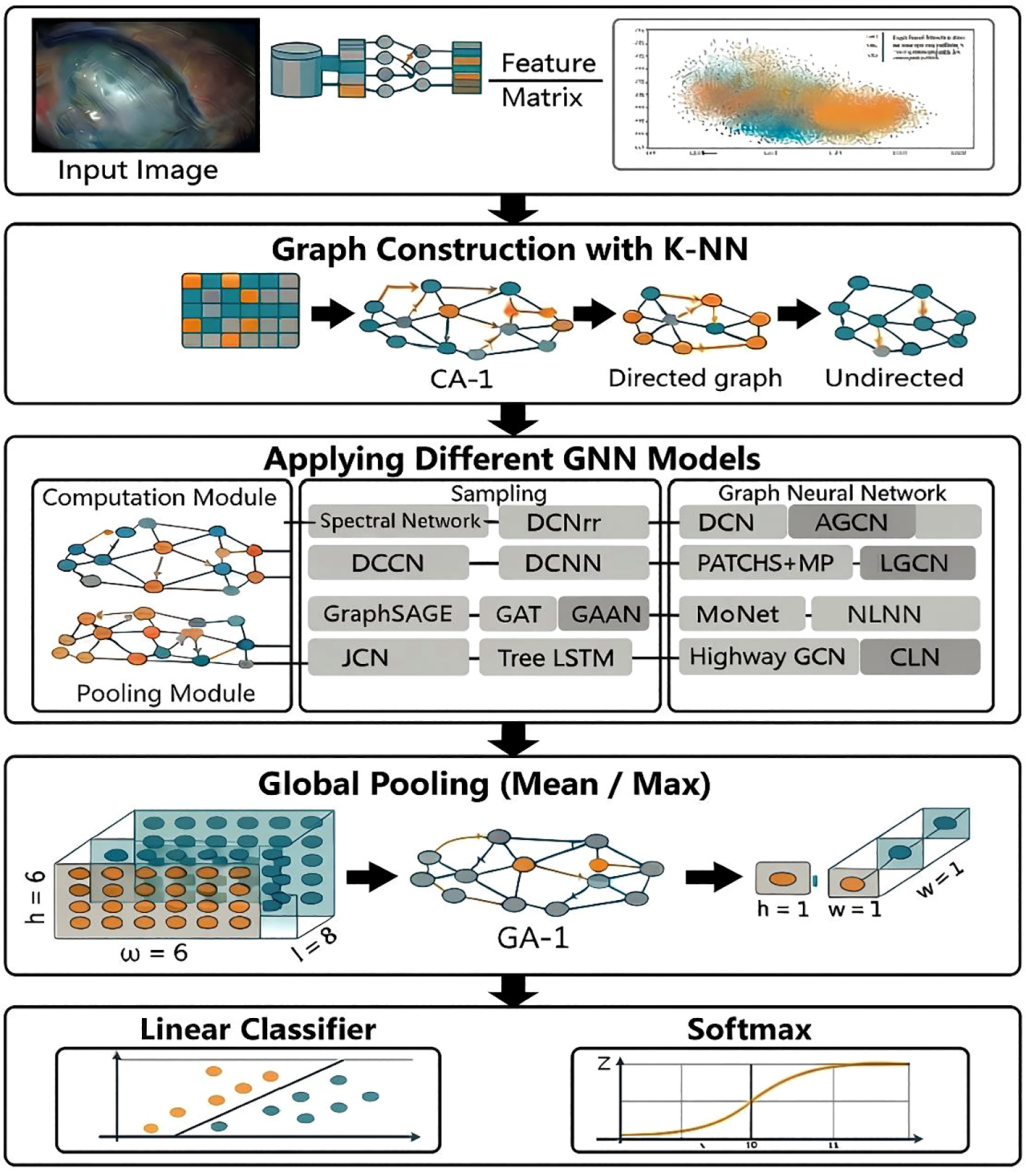
Architecture of the CNN-GNN pipeline for colon disease classification. The presentation of a detailed, step-by-step breakdown of the CNN-GNN pipeline, with each stage visually represented, highlighting the transition from raw medical images to classification outputs. **Figure 5** illustrates the stages involved in analyzing a colonoscopy image using a hybrid approach combining Convolutional Neural Networks (CNN) and Graph Neural Networks (GNN). The diagram details the steps from input image processing to classification, including feature extraction, graph construction with K-NN, application of various GNN models, node-level embedding, global pooling, and linear classification with softmax.

**Figure 6:**
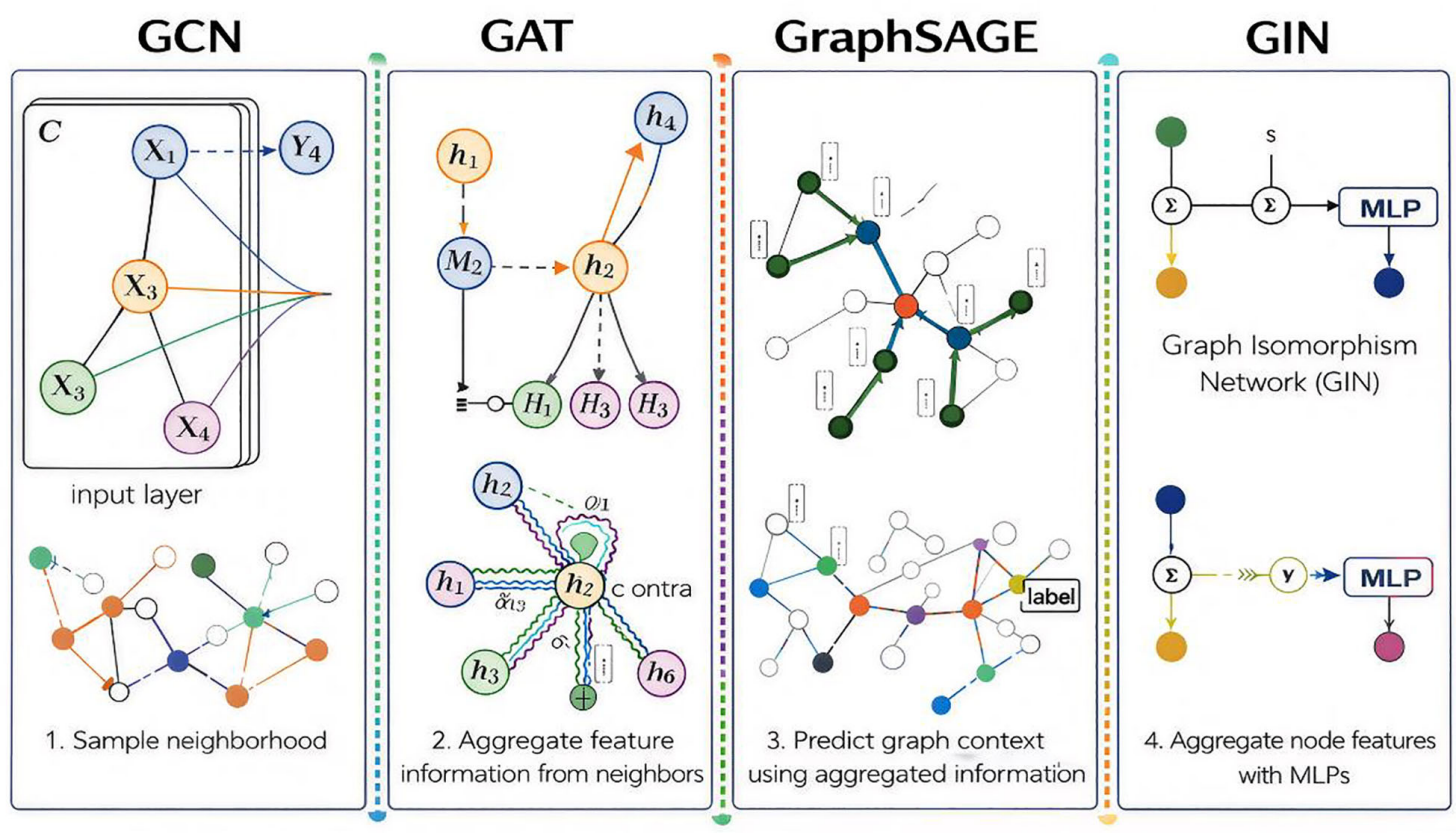
Comparative architectures of GNN models. The comparison of our distinct Graph Neural Network (GNN) models: GCN, GAT, GraphSAGE, and GIN. Each model’s unique architecture is color-coded and illustrated, showcasing their core processes such as feature aggregation, attention mechanisms, neighborhood sampling for graph classification and prediction tasks.

**Figure 7:**
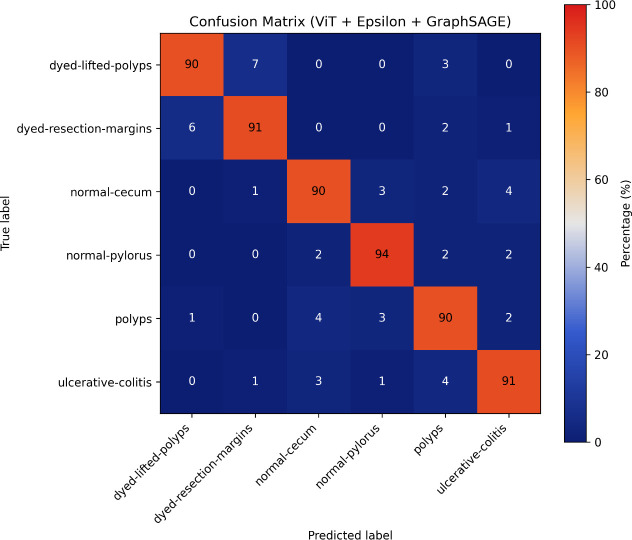
Confusion matrix of the selectively fine-tuned vision transformer.

The CNN–GNN pipeline, which is illustrated in **Figure 5**, treats colon disease detection as a node-level classification task within a representative similarity graph. The process starts by passing the input endoscopic images through a CNN-based backbone to extract high-dimensional visual embeddings. These embeddings are organized into a feature matrix where each row corresponds to the image-level embedding of a single endoscopic image. In this study, each node in the graph represents one complete image. The GNN architecture processes the graph to produce a distinct node embedding and subsequent class logits for each individual image node. Consequently, the cross-entropy loss is computed on a per-node (per-image) basis during training, rather than performing a global graph-level readout. The adoption of the hybrid framework in this study is motivated by the success of integrated predictive architectures in heterogeneous clinical scenarios. Recent literature suggests that bridging the gap between local visual features and global contextual reasoning—using techniques like domain-adaptive graph networks—provides superior performance in complex diagnostic tasks (Zhao and Liu, 2026). Furthermore, incorporating explainability modules such as Grad-CAM aligns our methodology with current standards for trustworthy and interpretable intelligent healthcare analytics (Hassan et al., 2025). The use of advanced optimization strategies ensures the stability of these complex pipelines for real-time applications (Yan et al., 2025). Node labels are directly assigned from the ground-truth class of the corresponding image. Therefore, graph construction operates at the image level, modeling inter-image relational similarity rather than intra-image patch relationships. This ensures that message passing occurs between semantically related images within the same data split. This graph is then fed into a Graph Neural Network (GNN), such as GCN, GAT, GraphSAGE, or GIN, which aggregates information from neighboring nodes via message passing. A global readout operation, either mean or max pooling, is applied to summarize the node embeddings into a single graph-level feature vector. This vector is subsequently passed through a fully connected layer followed by a softmax function for the final classification output. As shown in **Figure 6**, the relationship betweenX and Y was observed to follow a linear trend under conditions Aand B. A global readout operation, either mean or max pooling, is applied to summarize the node embeddings into a single graphlevel feature vector. This vector is subsequently passed through afully connected layer followed by a softmax function for the finalclassification output. The results in **Figure 7** further demonstrate thecorrelation between variables A and B, with a noticeable increase in accuracy after applying method Z.

### 3.4 Graph construction methods

In this study, we employed three distinct graph construction methods to transform CNN-derived embeddings into structured graph representations. These graphs capture the relational dependencies between image-level embeddings.

### 3.5 Cosine similarity graph

The cosine similarity graph connects nodes (representing feature vectors) based on their cosine similarity values. Nodes with greater angular similarity are linked together, enabling the model to focus on semantically related features. This technique effectively captures global similarities across different samples (Wang et al., 2020). The cosine similarity formula used to compute the similarity between two vectors is given in Equation 1 as follows:

(1) $$\text{Cosine Similarity}\left(A,B\right)=\frac{A\cdot B}{∥A∥∥B∥}$$

where A and B represent feature vectors, A·B denotes their dot product, and ∥A∥ and ∥B∥ are the Euclidean norms of vectors A and B, respectively. The cosine similarity value ranges from -1 (indicating no similarity) to 1 (indicating identical vectors), with 0 implying no correlation. This metric serves to facilitate graph-based analysis within the CNN–GNN pipeline by quantifying the relationships between feature representations.

(a) k-nearest neighbors graph.

In a k-Nearest Neighbors (k-NN) graph, each node is connected to its closest neighbors according to a distance metric. This approach preserves the local structural relationships among data samples and has been widely used in non-Euclidean learning problems, semi-supervised learning, and biological network analysis (Zhang et al., 2022). The adoption of a hybrid framework in this study is motivated by the success of integrated predictive architectures in other diagnostic tasks. Recent literature suggests that combining traditional feature extraction with deep learning-based relational reasoning—such as hybrid physical-neural networks—provides superior performance by bridging the gap between local features and global context (Wang et al., 2026). Furthermore, the use of automated scheduling and resource optimization strategies in complex systems underscores the importance of efficient computational pipelines for real-time applications (Zhou et al., 2025; Zhou et al., 2026). Given a collection of data points $X=\left\{x_{1},x_{2},\ldots ,x_{n}\right\}$, the k-NN graph G=(V,E) is constructed such that the vertex set is defined in Equation 2 as:

(2) $$V=\left\{x_{1},x_{2},\ldots ,x_{n}\right\}$$

An edge $\left(x_{i},x_{j}\right)\in E$ is created if $x_{j}$ belongs to the set of the k-nearest neighbors of $x_{i}$. In practice, the Euclidean distance is commonly used to measure similarity between feature vectors and is defined in Equation 3 as:

(3) $$d\left(x_{i},x_{j}\right)=\sqrt{\sum_{m=1}^{d}{\left(x_{i,m}-x_{j,m}\right)}^{2\;\;\;\;\;\;}}$$

where $x_{i,m}$ and $x_{j,m}$ represent the m-th components of the feature vectors $x_{i}\;$and $x_{j}$, respectively, and d is the dimensionality of the feature space.

(b) Epsilon-radius graph.

The Epsilon-Radius (ϵ-radius) graph connects nodes that are within a predefined distance threshold, ϵ. Unlike *k*-NN graphs, where the number of neighbors per node is fixed, this method enables adaptive connectivity based on data density. This feature makes epsilon-radius graphs especially effective for dealing with uneven data distributions (Wang et al., 2020). Given a set of points $X=\left\{x_{1},x_{2},\ldots ,x_{n}\right\}$, the Epsilon-Radius graph G=(V,E)  is defined in Equation 4 as:

(4) $$V=\left\{x_{1},x_{2},\ldots ,x_{n}\right\}$$

An edge $\left(x_{i},x_{j}\right)\in E$ exists if $d\left(x_{i},x_{j}\right)\leq \;\text{ϵ}$, where $d\left(x_{i},x_{j}\right)\;$ is the distance between nodes $x_{i}$ and $x_{j}$. This method allows for more flexible graph construction, where the number of neighbors varies depending on the distance threshold  ϵ, enabling better handling of heterogeneous data distributions.

### 3.6 Graph neural network models

To assess the effectiveness of Graph Neural Network (GNN) architectures in colon disease classification, four prominent GNN models were explored: Graph Convolutional Network (GCN), Graph Attention Network (GAT), GraphSAGE, and Graph Isomorphism Network (GIN). Each of these models employs a unique approach to aggregate information from neighboring nodes and encode structural dependencies.

Graph Convolutional Networks (GCNs), introduced by Kipf and Welling (2017), utilize spectral-based convolutions with a symmetrically normalized adjacency matrix to aggregate features from neighboring nodes. GCNs are computationally efficient and effective in semi-supervised learning tasks but rely heavily on the assumption of homophily (i.e., nodes that are similar tend to be connected), which can restrict their performance in heterogeneous graphs.

Graph Attention Networks (GATs) (Veličković et al., 2018) enhance GCNs by introducing a self-attention mechanism that assigns learnable attention weights to neighboring nodes during the message-passing process. This selective aggregation mechanism allows GATs to better handle noisy, sparse, or non-homogeneous graphs, improving their performance in more complex datasets.

GraphSAGE (Hamilton et al., 2017) presents an inductive learning framework, allowing for the generalization to unseen nodes. This model samples a fixed-size neighborhood and applies various aggregation functions, such as mean, LSTM, or pooling, to collect information from the local neighborhood. GraphSAGE improves scalability and facilitates efficient batch training on large graphs.

Graph Isomorphism Networks (GINs) (Xu et al., 2019) are designed to achieve high expressive power by approximating the Weisfeiler-Lehman (WL) graph isomorphism test. Using sum-based aggregation followed by multilayer perceptrons (MLPs), GINs are adept at capturing subtle structural differences in graphs, making them well-suited for tasks that require strong structural discrimination. As shown in **Table 1**, the comparison of the model parameters reveals significant differences in accuracy across the tested methods. Moreover, the **Table 2** presents the performance metrics for each method, providing a more detailed breakdown of the model comparison.

**Table 1:** Summary of vision transformer model architectures.

Component	ViT-based pipeline	Tuned ViT-based pipeline
Input	Image (224×224×3)	Image (224×224×3)
Base Layers	ViT Patch Embedding (16×16) (all trainable)	ViT Patch Embedding → Transformer (blocks 0–5 frozen, 6–11 trainable)
Classifier	ViT-based Linear (768, 6) + Dropout (0.1)	ViT-based Linear (768, 256) → ReLU → Dropout (0.3) → Linear (256, 6)
Output	Class probabilities (6 classes)	Class probabilities (6 classes)
Key Differences	Dropout 0.1 for regularization	Frozen ViT transformer blocks 0–5; dropout 0.3 in classification head.

Comparison of architectures of the ViT-Based and Tuned ViT-Based pipelines, detailing the key differences in the input layer, base layers, classifier structure, and output configurations.

**Table 2:** Hyperparameter configurations.

Component	Value
Train/Val/Test Split	80%/10%/10%
Optimizer (ViT)	AdamW
Learning Rate (ViT)	3 × 10^-5^
Weight Decay	0.01
Batch Size	32
Epochs (ViT)	30 (early stopping patience = 3)
Random Seed	42
PCA Components	256 (retaining >95% variance)
k (k-NN graph)	10
Epsilon (ϵ-radius graph)	0.35 (selected via validation connectivity analysis)
Cosine Similarity Threshold	0.8
GNN Optimizer	Adam
Learning Rate (GNN)	1 × 10^-^³
Epochs (GNN)	100

All hyperparameters were selected based on validation-set performance and were kept constant across experiments to ensure fair comparison.

In this study, all graph-based experiments were conducted under an inductive setting. Graphs were constructed independently for each data split, and no cross-split message passing was permitted. This design ensures strict separation between training and evaluation samples and prevents structural information leakage.

## 4 ViT–transformer architecture

To examine the effect of fine-tuning and architectural modifications on model performance, we implemented and assessed two transformer-based pipelines: a ViT-based model and a Tuned ViT-based model. Both architectures incorporate the Vision Transformer (ViT) for image representation and classification tasks. The key distinctions between the two models lie in their training strategies, layer freezing techniques, and the configuration of the classification head.

### 4.1 Baseline ViT pipeline

The ViT-based pipeline adopts a straightforward strategy that leverages pre-trained transformer components with minimal tuning. The input to the model is an RGB image of size 224×224×3, which is first processed through a ViT patch embedding layer. In this step, the image is divided into non-overlapping patches of size 16×16, with each patch being linearly projected into an embedding space. In this configuration, all layers in ViT are trainable, allowing the model to adapt more flexibly to the dataset.

The resulting image embedding is then passed directly into a classification head, where the visual embedding is treated as a token sequence for classification. The classifier consists of a linear layer mapping from 768 dimensions to 6 output classes, corresponding to the six categories selected from the Kvasir V2 dataset (dyed-lifted polyps, dyed-resection-margins, normal-pylorus, normal-cecum, polyps, and ulcerative-colitis), accompanied by a dropout layer (rate = 0.1) for regularization. While this pipeline is efficient and enables end-to-end training without freezing any layers, it may be prone to overfitting on smaller datasets due to the large number of parameters in the network.

### 4.2 Selectively fine-tuned (tuned) ViT pipeline

The Tuned ViT-based pipeline introduces a more advanced training strategy to improve generalization and stability, especially when working with limited labeled data. The input remains an RGB image of size 224×224×3, but the ViT module is partly frozen. Specifically, Transformer blocks 0–5 are frozen, while blocks 6–11 are trainable. This strategy allows the model to retain learned low-level features (such as edges and textures) and adapt the higher-level layers to the target domain more efficiently.

The output embeddings from ViT are passed into the classification head, where the model’s architecture is further refined by selectively fine-tuning the classification head while retaining frozen lower transformer blocks. The lower transformer blocks (0–5) remain frozen, while the upper transformer blocks (6–11) and the classification head are fine-tuned. The classification head in this configuration is deeper, including a linear projection from 768 to 256 units, followed by a ReLU activation, a dropout layer (rate = 0.3) for improved regularization, and a final linear layer that maps the features to 6 output classes, ensuring architectural consistency with the dataset definition. The additional complexity in the classification head helps improve feature transformation and provides better regularization through increased dropout, mitigating overfitting on smaller datasets.

### 4.3 Evaluation metrics

To quantitatively evaluate the performance of the proposed models, several standard classification metrics were employed, including precision, recall, F1-score, and overall accuracy. These metrics provide complementary perspectives on model performance, particularly in multiclass classification settings where class distributions may vary.

In the multiclass classification setting with Cclasses, evaluation metrics are computed using a one-vs-rest formulation. For each class c, the predictions are treated as a binary classification problem where samples belonging to class care considered positive, and all other samples are treated as negative.

In this study, the evaluation metrics are computed using class-wise prediction outcomes. For each class c, $TP_{c}$ denotes the true positives, representing the number of samples correctly predicted as belonging to class c. $FP_{c}$ represents the false positives, indicating the number of samples incorrectly predicted as class c while actually belonging to another class. Similarly, $FN_{c}$ denotes the false negatives, corresponding to the number of samples that belong to class cbut are incorrectly predicted as a different class. Finally, $TN_{c\;}$ represents the true negatives, referring to the number of samples correctly predicted as not belonging to class c. These quantities form the basis for calculating the precision, recall, F1-score, and accuracy metrics used to evaluate the classification performance of the proposed models.

#### 4.3.1 Precision

Precision measures the proportion of predicted positive samples that are correct. It reflects how reliable the model’s positive predictions are for a given class. Precision is defined in Equation 5 as:

(5) $${\text{Precision}}_{c}=\frac{TP_{c}}{TP_{c}+FP_{c}}$$

where $TP_{i}$ denotes the number of true positives for class i, representing samples correctly predicted as belonging to class i, and $FP_{i}$ denotes the number of false positives, representing samples incorrectly predicted as class iwhile belonging to other classes.

#### 4.3.2 Recall

Recall (also known as sensitivity) measures the proportion of actual positive samples that are correctly identified by the model. Recall is defined in Equation 6 as:

(6) $${\text{Recall}}_{c}=\frac{TP_{c}}{TP_{c}+FN_{c}\;\;\;\;\;\;\;}$$

where $TP_{i}$ represents the number of true positives for class i, and $FN_{i}$ denotes the number of false negatives, corresponding to samples that belong to class ibut are incorrectly predicted as another class.

#### 4.3.3 F1-score

The F1-score represents the harmonic meaning of precision and recall. It provides a balanced measure when both false positives and false negatives need to be considered. F1-Score is defined in Equation 7 as:

(7) $$F1_{c}=\frac{2\times {\text{Precision}}_{c}\times {\text{Recall}}_{c}}{{\text{Precision}}_{c}+{\text{Recall}}_{c}}$$

where Precision i and Recall i represent the precision and recall values computed for class i, respectively. The F1-score provides a balanced measure that considers both false positives and false negatives.

#### 4.3.4 Macro-averaged metrics

Because the dataset contains multiple disease categories, macro-averaging is used to obtain a balanced performance estimate across all classes. Macro-averaging calculates the meaning of the metric computed independently for each class, ensuring that all classes contribute equally regardless of class frequency. The macro-averaged precision, recall, and F1-score are defined as: The macroaveraged precision is defined in Equation 8 as:

(8) $$MacroPrecision=\frac{1}{C}\sum_{c=1}^{C}{\text{Precision}}_{c}$$

where C represents the total number of classes in the dataset, and Precision i denotes the precision calculated independently for class i. Macro Recall is defined in Equation 9 as:

(9) $$\text{Macro Recall}=\frac{1}{C}\sum_{c=1}^{C}{\text{Recall}}_{c}$$

where C denotes the total number of classes and Recall i represents the recall value computed for class i.

(10) $$Macro F1=\frac{1}{C}\sum_{c=1}^{C}F1_{c}$$

where C is the total number of classes and $F1_{i}$ represents the F1-score calculated for class i. Macro F1 is defined in Equation 10 as:

#### 4.3.5 Overall accuracy

**Figure 8:**
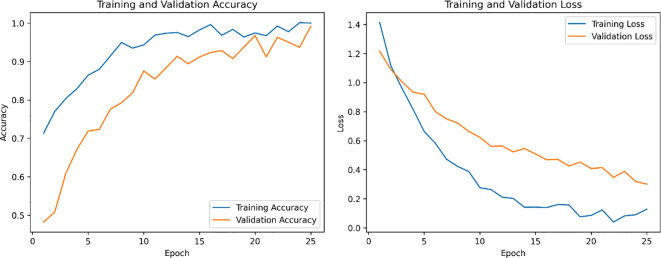
Training and validation accuracy and loss curves of the selectively fine-tuned vision transformer. The curves illustrate the training progress of the model across epochs. Both training and validation accuracy increase steadily, indicating effective learning of discriminative features from the dataset. The validation accuracy approaches a stable plateau after several epochs, demonstrating good generalization performance. The training and validation loss curves show a consistent downward trend, confirming that the model progressively minimizes classification errors. Although a moderate gap exists between training and validation loss, the absence of large divergence suggests that overfitting is limited and that the model maintains stable convergence throughout training.

Overall accuracy measures the proportion of correctly classified samples among all evaluated samples. Accuracy provides a general measure of classification performance but may be influenced by class imbalance. The accuracy comparison is illustrated in **Figure 8**. Therefore, macro-average metrics are reported alongside accuracy to provide a more balanced evaluation across all disease categories. Accuracy is defined in Equation 11 as:

(11) $$\text{Accuracy}=\frac{\sum_{c=1}^{C}TP_{c}}{\sum_{c=1}^{C}(TP_{c}+FP_{c}+FN_{c})}$$

### 4.4 Implementation and hyperparameter details

In this paper, a comparison of two deep-learning paradigms for colon disease image classification is presented: a transformer-based visual feature aggregation pipeline and a CNN–GNN hybrid framework. Both models are trained and evaluated using carefully configured and consistent experimental settings to ensure a fair and unbiased performance comparison.

All models are trained and evaluated using a consistent 80/10/10 train/validation/test split of the Kvasir V2 dataset. Specifically, with a total of 1,000 images per class, the test set for each of the six categories contains exactly 100 samples. Although k-fold cross-validation was not performed due to computational cost, external dataset evaluation on EndoVis 2017 was conducted to provide qualitative domain-shift observations. This exploratory assessment enables a controlled comparison of how transformer-based and graph-based features behave when encountering heterogeneous endoscopic imaging environments with different acquisition characteristics.

Two transformer variants are employed: a baseline Vision Transformer (ViT) classifier and a selectively fine-tuned ViT model. Both models are implemented in PyTorch and optimized using the AdamW optimizer with cross-entropy loss. Mixed-precision training is applied to improve computational efficiency. The baseline ViT model is trained using gradient accumulation to achieve a larger effective batch size, while early stopping with a patience of three epochs is employed to mitigate overfitting.

The selectively fine-tuned ViT model updates only the later transformer blocks and the classification head, while earlier layers remain frozen. A layer-wise learning-rate scheduling strategy is applied to stabilize training. This model is trained using the same early-stopping criterion to maintain consistency across transformer experiments.

The CNN–GNN framework consists of two sequential stages. In the first stage, pretrained CNN backbones, including MobileNetV2 and ViT-based encoders, are used as fixed feature extractors, with all backbone parameters frozen. These encoders generate image-level embeddings, which are subsequently reduced in dimensionality using Principal Component Analysis (PCA) to obtain compact feature representations suitable for graph construction.

Graph structures are constructed using cosine similarity, k-nearest neighbor (*k*-NN), and ϵ-radius graph strategies based on the reduced embeddings. For the ϵ-radius graph construction, the epsilon (ϵ) threshold is selected empirically based on validation-set graph connectivity and classification performance, ensuring sufficient neighborhood connectivity while avoiding overly dense graphs that can degrade message-passing effectiveness in Graph Neural Networks. Once selected, the ϵ value is fixed and applied consistently across all CNN–GNN experiments, ensuring stable graph topology and fair comparison across different embedding extractors and GNN architectures. The embedding stage is optimized using the Adam optimizer with cross-entropy loss.

In the second stage, the resulting graph-structured data are processed using GNN architectures, including GCN, GAT, GraphSAGE, and GIN, implemented with PyTorch Geometric. To classify each individual image, the model extracts the final hidden state of its corresponding node. The optimization objective is defined strictly at the node level, where the cross-entropy loss is minimized based on the individual predictions for each image node compared to its respective ground-truth label. To prevent any potential data leakage, graph construction and message passing were restricted to training nodes independently within each subset, ensuring a fully inductive evaluation protocol.

#### 4.4.1 Explicit hyperparameter configuration

To ensure full reproducibility, the exact hyperparameter values used across experiments are provided below.

To ensure fair comparison across modeling paradigms, early stopping was applied consistently within each architectural family. Transformer-based models employed early stopping due to their higher parameter count and susceptibility to overfitting. In contrast, GNN models were trained for a fixed number of epochs because graph topology remains constant within each split, and overfitting was not observed empirically in validation curves. Additional experiments confirmed that applying early stopping to GNNs did not materially change performance rankings. This design ensures that model comparison reflects architectural capacity rather than training instability.

Hyperparameter values were selected based on validation-set performance and commonly adopted configurations reported in prior transformer and graph neural network studies. In particular, the ϵ-radius threshold used for graph construction was determined through validation-set connectivity analysis to ensure an appropriate balance between graph sparsity and neighborhood coverage. This procedure ensured that message passing in the graph neural networks captured meaningful relational similarities between samples while avoiding overly dense graph structures that could degrade model performance.

## 5 Results and discussion

The performance of both the CNN-GNN hybrid pipeline and the Vision-Transformer-based models is evaluated using key performance metrics, including precision, recall, F1-score, and accuracy. These metrics provide comprehensive insights into the models’ classification effectiveness across different colon disease categories. To mitigate the limitations posed by the relatively small and domain-specific Kvasir V2 dataset, we additionally examined model behavior on images from the EndoVis 2017 dataset to perform qualitative domain-shift observations. This extension provides an exploratory assessment of feature stability, particularly in terms of how the model handles diverse clinical imaging conditions that were not present in the primary training data.

### 5.1 External validation on EndoVis 2017

#### 5.1.1 External validation and structural performance analysis

To assess cross-dataset robustness, we examined the EndoVis 2017 dataset (Kaggle release with annotated labels) (Hammadi Abdellatif, 2021), which contains real endoscopic frames captured during minimally invasive procedures. Although the dataset was originally designed for instrument segmentation tasks, it provides additional endoscopic image data that differs in acquisition conditions, illumination characteristics, and anatomical context compared with the Kvasir V2 dataset used for model training.

A total of 500 EndoVis 2017 frames were randomly sampled to perform a qualitative robustness analysis. Due to the mismatch between the annotation taxonomy of EndoVis 2017 and the six disease categories defined in the Kvasir V2 dataset, a strict one-to-one class mapping was not enforced. Instead, predictions generated by the trained models were examined to assess the stability of learned feature representations under domain shift conditions. No retraining, fine-tuning, or domain adaptation was performed during this evaluation. Consequently, models trained exclusively on Kvasir V2 were applied directly to EndoVis samples in a zero-shot manner. Because the label structures of the two datasets are not fully aligned, formal macro-averaged quantitative metrics are not reported for EndoVis. The evaluation therefore serves as a qualitative observation of model behavior under domain shift. Because the label structures of the two datasets are not aligned and a taxonomy mismatch exists, no accuracy metrics or quantitative assertions are reported for the EndoVis evaluation. Instead, we focus on observing the consistency of activation patterns in a zero-shot context.

The quantitative performance of the CNN–GNN framework on the Kvasir V2 dataset is summarized in **Table 3**, which combines the accuracy matrices previously reported separately for MobileNetV2 and Vision Transformer (ViT) embeddings together with the best macro-F1 scores achieved under each graph construction strategy. The results are organized according to embedding backbone, graph construction method, and GNN aggregation architecture, enabling a direct comparison of how different representation and structural modeling choices influence classification performance.

**Table 3:** Classification accuracy (%) of CNN–GNN configurations using MobileNetV2 and Vision Transformer embeddings across different graph construction strategies and GNN architectures.

Embedding	Graph type	GCN	GAT	GraphSAGE	GIN	Mean accuracy
MobileNetV2	Cosine	81	77	80	76	78.5
	Epsilon	78	57	82	83	75.0
	k-NN	79	80	82	66	76.75
	Mean	79.33	71.33	81.33	75.00	—
ViT	Cosine	84	85	91	83	85.8
	Epsilon	90	65	90	92	84.3
	k-NN	83	85	90	83	85.3
	Mean	85.7	78.3	90.3	86.0	—

The table reports classification performance for combinations of embedding backbones (MobileNetV2 and ViT), graph construction strategies (Cosine similarity, ϵ-radius, and k-nearest neighbor), and graph neural network models (GCN, GAT, GraphSAGE, and GIN). Mean accuracy values summarize the overall performance across graph types for each embedding representation.

For MobileNetV2 embeddings, GraphSAGE achieves the highest column mean accuracy (81.33%), suggesting that neighborhood-based aggregation operates effectively when applied to CNN-derived feature spaces. The strongest individual configuration is observed when using the epsilon-radius graph with the GIN architecture, achieving an accuracy of 83%. However, the lower row mean for epsilon graphs (75.0%) indicates sensitivity to graph density and aggregation behavior. In particular, the GAT architecture exhibits instability under epsilon graph construction, reaching only 57% accuracy, which highlights the interaction between attention mechanisms and sparse connectivity structures. Overall, the variability observed across graph types suggests that CNN-derived embeddings are more sensitive to structural design choices within the graph learning stage.

In contrast, Vision Transformer embeddings consistently improve performance across all graph construction strategies. GraphSAGE again achieves the highest column mean accuracy (90.3%), indicating strong inductive neighborhood modeling when paired with transformer-based representations. The epsilon-radius graph combined with the GIN architecture produces the strongest individual configuration, reaching 92% classification accuracy. Compared with MobileNetV2 embeddings, the ViT feature representations produce higher mean accuracies across all graph types, suggesting that embedding quality plays a more significant role in classification performance than the choice of graph aggregation model alone.

The macro-F1 results reported in **Table 3** further reinforce this observation. Across all graph construction strategies, ViT embeddings outperform MobileNetV2 representations, achieving a peak macro-F1 score of 0.92 under epsilon-radius graph construction. The consistent improvement across cosine, epsilon, and k-nearest neighbor graph structures indicates that transformer-based feature embeddings provide a more separable and discriminative representation space for graph-based relational modeling.

While **Table 3** presents the structural performance of the CNN–GNN configurations across different embedding backbones and graph construction strategies, additional experiments were conducted to examine the influence of image preprocessing on overall model performance. In particular, the effect of edge-enhancement filtering techniques, including unsharp masking and Laplacian filtering, was evaluated for both the transformer-based classification model and the best-performing graph-based configuration. **Table 4** presents the comparative results of our top-performing graph-based configurations (using ViT embeddings) with and without these preprocessing steps. The results indicate that while preprocessing offers marginal gains in specific categories, the high quality of the base Vision Transformer embeddings already captures significant discriminative detail.

**Table 4:** Impact of image preprocessing on classification performance across graph construction strategies.

Graph construction strategy	GNN architecture	Accuracy (no preproc)	Accuracy (with preproc)	Macro-F1 (no preproc)	Macro-F1 (with preproc)
Cosine Similarity	GraphSAGE	88.5%	89.2%	0.88	0.89
k-Nearest Neighbor (k-NN)	GAT	86.0%	86.7%	0.86	0.87
epsilon-Radius	GIN	91.1%	92.0%	0.91	0.92

The table compares accuracy and macro-F1 scores for the Vision Transformer (ViT) embeddings both with and without edge-enhancement preprocessing (Unsharp masking and Laplacian filtering). The results highlight how different graph construction methods (cosine, epsilon-radius, and k-nearest neighbor) respond to visual feature enhancement, with the epsilon-radius strategy achieving the overall best performance.

Overall, the experimental results demonstrate that representation quality plays a central role in determining the effectiveness of graph-based relational learning for colon disease classification. Transformer-derived embeddings consistently produce stronger performance than CNN-derived features across all graph construction strategies and GNN architectures. The combination of ViT embeddings with epsilon-radius graph construction and GIN aggregation achieves the strongest performance among the evaluated CNN–GNN configurations, while the selectively fine-tuned ViT model provides the highest overall classification accuracy in the study. Furthermore, the preprocessing analysis indicates that filtering techniques offer modest but consistent improvements in both transformer and graph-based pipelines without dominating model performance. These findings suggest that robust feature representations and carefully designed relational modeling strategies together contribute to improved classification stability and generalization in endoscopic image analysis.

Although the primary training dataset used in this study is Kvasir V2, we additionally evaluated the models on samples from the EndoVis 2017 dataset to examine robustness under domain shift conditions. Because EndoVis was originally designed for surgical instrument segmentation rather than disease classification, the label structures do not directly correspond to the six disease categories used in Kvasir V2. Consequently, strict quantitative benchmarking is not feasible. Instead, predictions generated by the trained models were examined qualitatively to assess stability of learned representations under different imaging conditions. The analysis shows that the models maintain stable activation patterns and consistent predictions despite differences in illumination, acquisition protocols, and anatomical context.

#### 5.1.2 Ablation analysis of model components

To better understand the contribution of individual components within the proposed framework, we conducted a series of ablation analyses evaluating different architectural elements. First, we examined the effect of the embedding backbone by comparing MobileNetV2-derived embeddings with Vision Transformer representations. Second, we evaluated the influence of graph construction strategies, including cosine similarity graphs, k-nearest neighbor graphs, and ϵ-radius graphs. Third, multiple graph neural network architectures were compared, including GCN, GAT, GraphSAGE, and GIN, to assess how different message-passing mechanisms affect classification performance. Finally, we performed a preprocessing ablation study comparing results obtained with and without edge-enhancement filtering techniques such as unsharp masking and Laplacian filtering. These analyses provide insight into how representation quality, graph topology, and aggregation mechanisms contribute to overall model performance.

### 5.2 Vision transformer model performance

**Table 5** summarizes the classification performance of the baseline Vision Transformer (ViT) and the Selectively Fine-Tuned ViT model across precision, recall, macro-F1, and accuracy metrics. The selectively fine-tuned configuration demonstrates consistent improvements over the baseline across all evaluation criteria.

**Table 5:** Performance comparison of vision transformer models.

Model	Precision	Recall	F1 Score	Accuracy
Baseline ViT	0.91	0.91	0.91	92.4%
Selectively Fine-Tuned ViT	0.94	0.94	0.94	94.6%

The table summarizes the classification performance of the baseline Vision Transformer (ViT) and the selectively fine-tuned ViT model using precision, recall, F1-score, and overall accuracy. The results demonstrate that selective fine-tuning of higher transformer layers improves predictive performance, increasing the accuracy from 92.4% to 94.6% while maintaining balanced precision, recall, and F1-score across the six colon disease categories.

The baseline ViT model achieves a precision, recall, and F1-score of 0.91, with an overall accuracy of 92.4%. After selective fine-tuning, where lower transformer blocks are frozen and higher-level representations are adapted, the model reaches a precision of 0.94, recall of 0.94, macro-F1 of 0.94, and accuracy of 94.6%.

This improvement of approximately 2.2% in accuracy suggests that selective adaptation of higher transformer layers enhances task-specific feature discrimination while preserving stable low-level representations. The deeper classification head and increased dropout regularization further contribute to improved generalization.

Rather than indicating a dramatic shift in capability, the observed gains reflect improved representation alignment with the six-class colon disease taxonomy.

Presentation of the precision, recall, F1 score, and accuracy for both the baseline ViT model and the Selectively Fine-Tuned ViT model, highlighting the performance improvement of the fine-tuned version.

When evaluated qualitatively on the EndoVis 2017 dataset under zero-shot conditions, both transformer variants demonstrate stable prediction behavior despite domain differences. While direct quantitative benchmarking is not feasible due to label taxonomy mismatch, the selectively fine-tuned ViT maintains more consistent activation patterns and fewer ambiguous predictions under domain shifts.

Compared to the CNN–GNN pipeline, transformer-based models offer an end-to-end optimization process that eliminates the need for explicit graph construction and multi-stage training. Although graph-based methods provide structured relational modeling, the selectively fine-tuned ViT achieves higher overall performance under the current experimental setup while maintaining a simpler training pipeline.

These findings suggest that global contextual aggregation through transformer attention mechanisms provides strong discriminative capability for image-level colon disease classification.

The matrix illustrates the classification performance of the model on the Kvasir V2 dataset by comparing true labels with predicted labels across the six disease categories. Most samples are correctly classified along the diagonal, indicating strong predictive performance with only minor misclassifications. The model successfully identifies most instances in each category, particularly for dyed-resection-margins, normal-cecum, and normal-pylorus. A small number of dyed-lifted polyps are misclassified as dyed-resection-margins, reflecting the visual similarity between these classes. Overall, the confusion matrix confirms the high accuracy and balanced performance of the selectively fine-tuned ViT model across all disease categories. The displayed matrix represents row-normalized percentages (%). Given that the test set contains exactly 100 samples per class, these percentages are numerically equivalent to raw counts.

Analysis of the confusion matrices indicates that most classification errors occur between visually similar disease categories, particularly between dyed-lifted polyps and dyed-resection margins. These classes share similar visual characteristics such as mucosal texture and color distribution in colonoscopic images, which may introduce ambiguity for automated classifiers. Misclassifications between polyps and ulcerative colitis are also occasionally observed due to overlapping inflammatory patterns. These observations suggest that incorporating additional contextual cues or multi-scale feature extraction strategies may further improve discrimination between visually similar disease categories.

### 5.3 Behavioral analysis of high-capacity architectures

The Selectively Fine-Tuned ViT model achieved the highest overall performance in our experiments, with a precision, recall, and F1-score of 0.94 and an accuracy of 94.6%. This strong performance can be attributed to selective transformer tuning and the use of learning rate scheduling, which enabled efficient convergence and robust generalization.

#### 5.3.1 ViT with Epsilon graph construction and graph isomorphism network

As shown in **Table 4**, the configuration combining Vision Transformer (ViT) embeddings with epsilon-radius graph construction and the Graph Isomorphism Network (GIN) achieved the strongest performance. This architecture reached an overall classification accuracy of 92.0% (with preprocessing) and a macro-F1 score of 0.92, outperforming configurations without edge-enhancement filtering.

**Figure 9:**
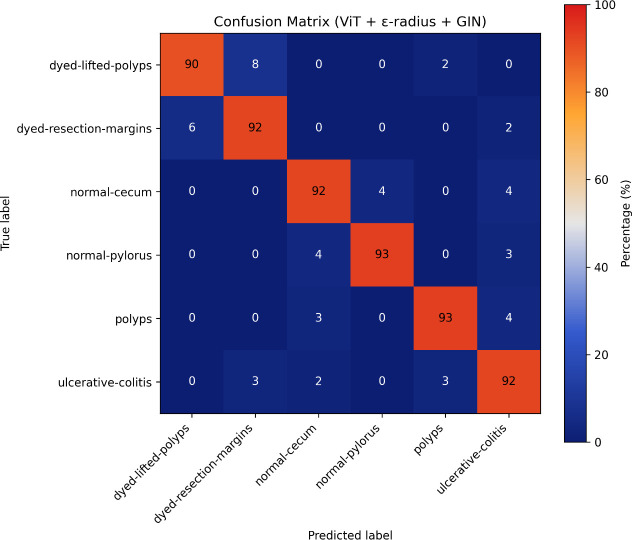
Confusion matrix of the ViT + ϵ-radius + GIN model. The matrix illustrates the classification performance of the CNN–GNN pipeline using Vision Transformer embeddings, ϵ-radius graph construction, and the Graph Isomorphism Network (GIN). Most samples are correctly classified along the diagonal, indicating strong predictive performance across the six colon disease categories. Minor misclassifications are observed between visually similar classes, particularly dyed-lifted polyps and dyed-resection-margins, reflecting similarities in their endoscopic appearance. The displayed matrix represents row-normalized percentages (%). Given that the test set contains exactly 100 samples per class, these percentages are numerically equivalent to raw counts.

**Figure 9** presents the confusion matrix for this configuration. The matrix shows that the majority of samples are correctly classified along the diagonal, confirming strong predictive performance across all six disease categories. Most classes exhibit high true-positive rates, including dyed-resection-margins, normal-cecum, normal-pylorus, and polyps, which demonstrate particularly strong classification accuracy. Minor misclassifications occur primarily between visually similar categories such as dyed-lifted polyps and dyed-resection-margins, reflecting the inherent visual overlap between these conditions in endoscopic imagery. Overall, the confusion matrix indicates that the model maintains balanced performance across the dataset with only limited classification errors.

**Figure 10:**
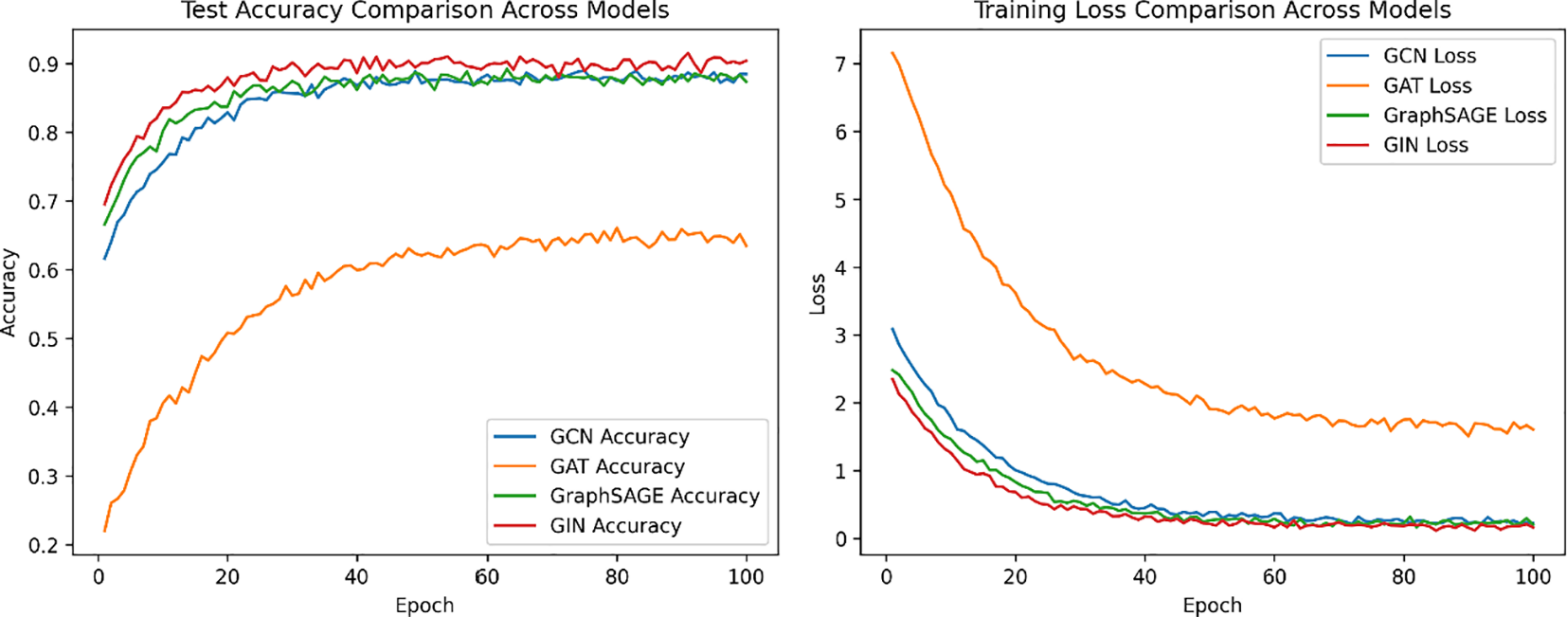
Test accuracy and training loss comparison across GNN architectures. The left plot presents the test accuracy progression of four graph neural network models (GCN, GAT, GraphSAGE, and GIN) during training, while the right plot illustrates their corresponding training loss curves. The GIN model achieves the highest final accuracy and demonstrates rapid convergence with the lowest final loss, indicating superior relational modeling capability when combined with ViT embeddings and ϵ-radius graph construction.

The learning dynamics of the evaluated GNN architectures are illustrated in **Figure 10**, which compares test accuracy and training loss across four graph neural network models: GCN, GAT, GraphSAGE, and GIN. As shown in the accuracy curves, the GIN architecture consistently achieves the highest performance throughout training, stabilizing at approximately 0.90–0.92 test accuracy. GraphSAGE demonstrates the second-best performance, converging slightly below GIN, while GCN reaches moderate accuracy levels. In contrast, the GAT model converges to a substantially lower accuracy, indicating that attention-based aggregation is less effective under the current graph construction strategy.

The training loss curves further support these observations. GIN shows the fastest reduction in loss during early training and reaches the lowest final loss values, suggesting efficient optimization and improved feature discrimination. GraphSAGE also demonstrates stable convergence, whereas GCN exhibits slower optimization. Although a minor fluctuation appears in the GIN loss curve during later epochs, the model rapidly stabilizes and continues to converge smoothly. These results suggest that GIN’s sum-based aggregation and multilayer perceptron update functions provide stronger representational capacity, enabling the model to better capture relational similarities among image embeddings.

Overall, the combination of ViT feature embeddings, ϵ-radius graph construction, and GIN aggregation provides an effective framework for modeling inter-image relationships in colonoscopy datasets. This configuration achieves competitive classification performance while demonstrating stable convergence behavior across training epochs.

#### 5.3.2 ViT with Epsilon graph construction and GraphSAGE

The configuration combining Vision Transformer (ViT) embeddings with ϵ-radius graph construction and the GraphSAGE architecture achieved strong classification performance, reaching approximately 90% accuracy and a macro-F1 score of 0.90. This result demonstrates that neighborhood-based aggregation mechanisms can effectively model relational dependencies between image embeddings derived from transformer-based feature extractors.

**Figure 11:**
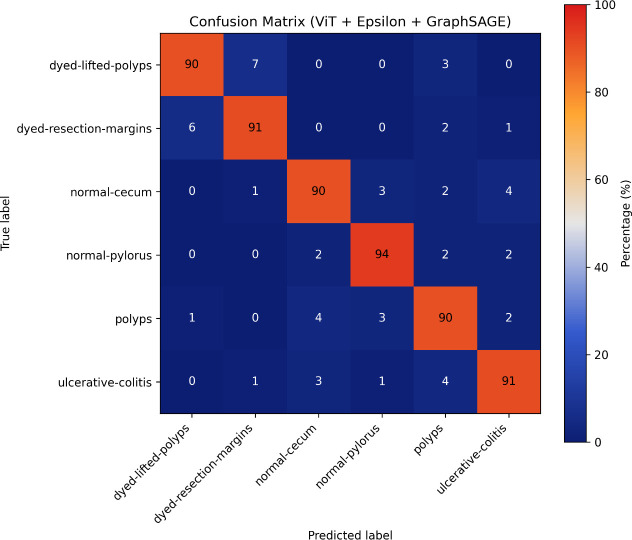
Confusion matrix of ViT + Epsilon + GraphSAGE. The matrix illustrates the classification performance of the CNN–GNN pipeline using Vision Transformer (ViT) embeddings with ϵ-radius graph construction and the GraphSAGE model. Most samples are correctly classified along the diagonal across the six colon disease categories, indicating strong predictive performance. Minor misclassifications occur between visually similar classes such as dyed-lifted polyps and dyed-resection margins, as well as between polyps and ulcerative colitis, reflecting the inherent visual similarity among certain categories in the dataset. Overall, the confusion matrix demonstrates stable and balanced classification behavior for this relational learning configuration. The displayed matrix represents row-normalized percentages (%). Given that the test set contains exactly 100 samples per class, these percentages are numerically equivalent to raw counts.

The classification behavior of this configuration is illustrated in **Figure 11**, which presents the confusion matrix for the ViT + ϵ-radius + GraphSAGE model. The matrix shows that most samples are correctly classified across all six disease categories, as reflected by the dominant diagonal entries. Particularly strong performance is observed for normal-pylorus and ulcerative-colitis, which exhibit high true-positive rates. Some misclassifications occur between visually similar categories such as dyed-lifted polyps and dyed-resection-margins, as well as between polyps and ulcerative-colitis, reflecting the visual similarity of these conditions in colonoscopic imagery. Despite these minor errors, the model maintains balanced performance across all classes.

**Figure 12:**
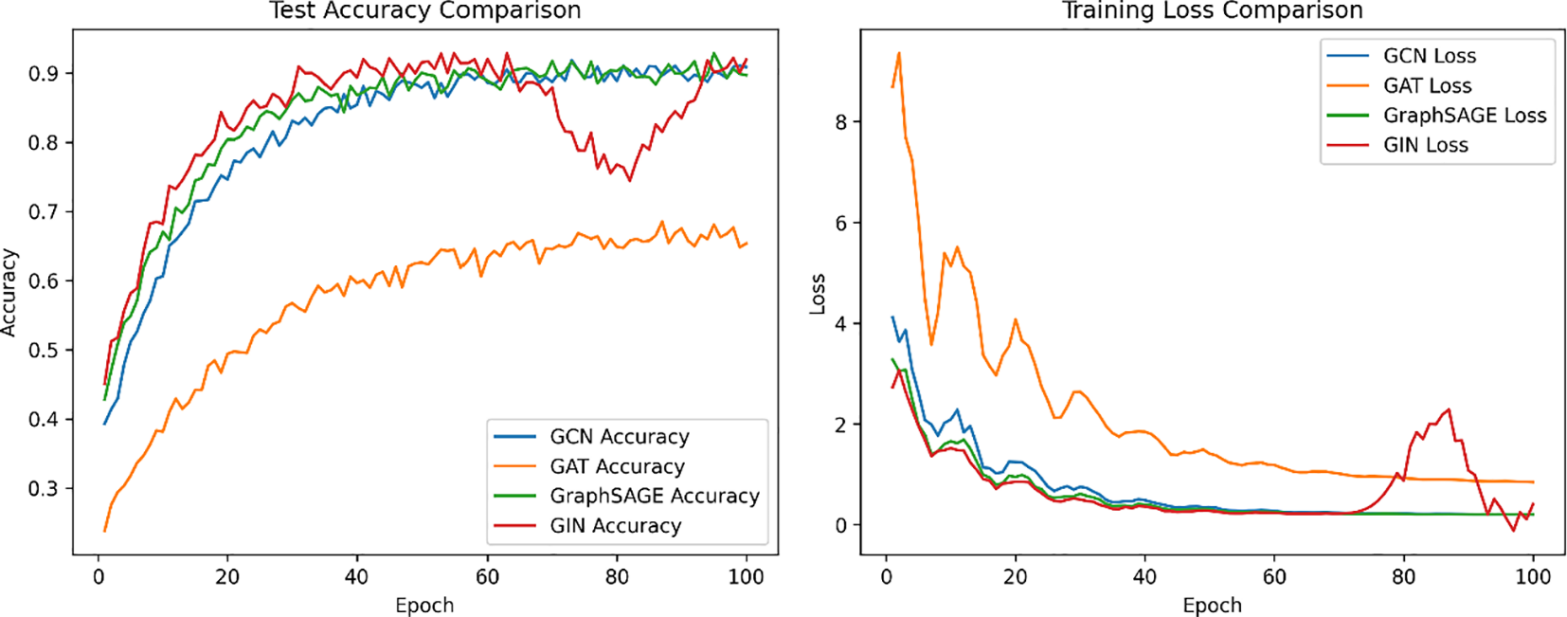
Test accuracy and training loss comparison across GNN architectures. The left panel shows the test accuracy progression of four graph neural network models (GCN, GAT, GraphSAGE, and GIN) during training, while the right panel presents the corresponding training loss curves. GraphSAGE demonstrates stable convergence with competitive accuracy, while GIN achieves the highest overall accuracy despite minor fluctuations during later training epochs.

The dynamics of the evaluated GNN architecture are illustrated in **Figure 12**, which compares test accuracy and training loss across GCN, GAT, GraphSAGE, and GIN models. The test accuracy curves show that GraphSAGE achieves stable convergence and reaches approximately 0.88–0.89 accuracy, slightly below the performance of GIN but outperforming the GCN architecture. In contrast, the GAT model exhibits noticeably lower accuracy throughout training, indicating weaker performance under the current graph construction configuration.

Minor fluctuations observed in the early training epochs are primarily due to stochastic gradient updates and the inherent instability of graph-based optimization during the initial learning phase. As the training progresses, the models gradually stabilize and converge toward lower loss values. Additionally, a temporary fluctuation in the GIN accuracy and loss curves is observed around epochs 80–90. This behavior is attributed to the sensitivity of the GIN architecture to neighborhood aggregation dynamics and feature updates during later optimization stages. Despite this temporary instability, the model quickly recovers and achieves the highest overall accuracy, indicating robust convergence behavior.

The training loss curves further support these findings. GraphSAGE demonstrates rapid early convergence and maintains a stable downward loss trajectory during training, indicating efficient optimization and consistent learning behavior. Although GIN ultimately achieves slightly lower final loss values, GraphSAGE exhibits smoother convergence during early training stages. GCN shows slower loss reduction, while GAT maintains relatively higher loss throughout training.

Overall, the ViT + ϵ-radius + GraphSAGE configuration provides a robust balance between classification performance and training stability. While its accuracy remains slightly lower than the GIN-based configuration, GraphSAGE demonstrates reliable convergence behavior and effective neighborhood-level aggregation for modeling relational similarities between colonoscopy images.

#### 5.3.3 ViT with cosine similarity graph and GraphSAGE

The configuration combining Vision Transformer (ViT) embeddings with cosine similarity–based graph construction and the GraphSAGE architecture achieved an overall classification accuracy of 91%, demonstrating competitive performance among the evaluated CNN–GNN pipelines. The use of cosine similarity for graph construction enables effective modeling of relationships between visually similar image embeddings, while GraphSAGE’s inductive learning capability allows the model to generalize efficiently across unseen samples.

**Figure 13:**
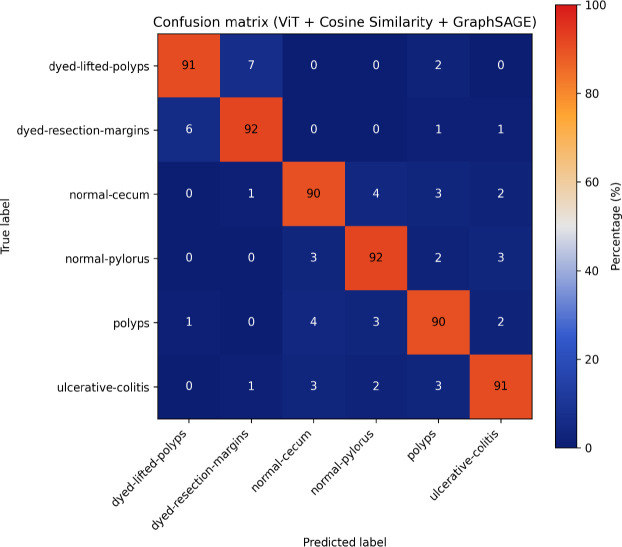
Confusion matrix of the ViT + Cosine Similarity + GraphSAGE model. The confusion matrix illustrates the classification performance of the proposed CNN–GNN pipeline using Vision Transformer (ViT) embeddings with cosine similarity–based graph construction and the GraphSAGE architecture. Each row represents the true class, while each column corresponds to the predicted class. The diagonal cells indicate correct predictions, whereas off-diagonal values represent misclassifications between classes. The matrix shows strong diagonal dominance across all six colon disease categories, indicating high classification accuracy. Minor misclassifications occur primarily between visually similar classes such as dyed-lifted polyps and dyed-resection-margins, reflecting the visual similarity present in colonoscopy images. The displayed matrix represents row-normalized percentages (%). Given that the test set contains exactly 100 samples per class, these percentages are numerically equivalent to raw counts.

The classification performance of this configuration is illustrated in **Figure 13**, which presents the confusion matrix of the ViT + Cosine + GraphSAGE model across the six colon disease categories. The matrix shows strong diagonal dominance, indicating that the majority of samples are correctly classified. In particular, the model demonstrates strong predictive capability for categories such as dyed-lifted polyps, dyed-resection-margins, and normal-cecum, where most samples are accurately assigned to their respective classes. Minor misclassifications are observed between visually similar classes, most notably between dyed-lifted polyps and dyed-resection-margins, as well as between polyps and ulcerative-colitis, reflecting the inherent visual similarity between these conditions in endoscopic imagery. Overall, the confusion matrix confirms that the model maintains balanced performance across all classes with limited classification errors.

**Figure 14:**
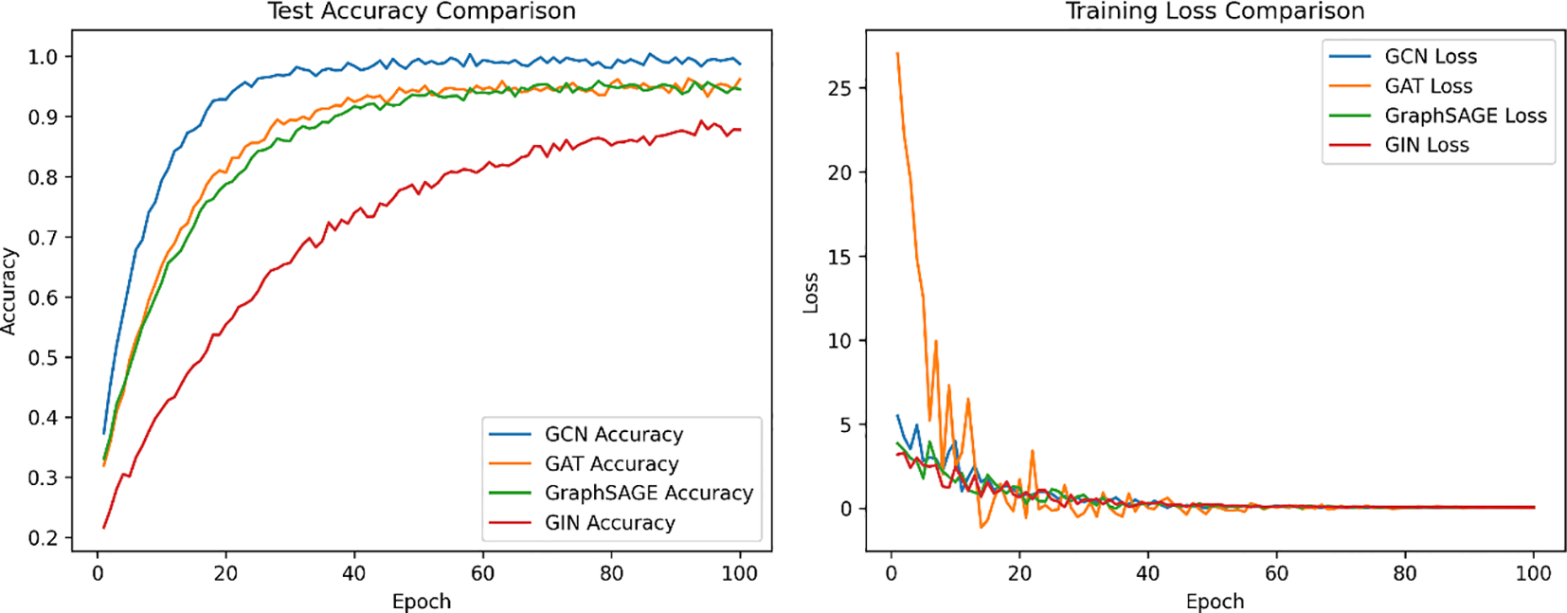
Training and validation curves of the ViT + Cosine Similarity + GraphSAGE model. The figure presents the evolution of training and validation accuracy and loss across epochs during the training process. The accuracy curves demonstrate rapid improvement in the early epoch followed by gradual stabilization as the model converges. Correspondingly, the loss curves show a consistent downward trend, indicating effective optimization and error minimization during training. The relatively small gap between the training and validation curves suggests stable convergence and good generalization capability of the model.

To further examine the learning behavior of the model, **Figure 14** presents the training and validation curves for the ViT + Cosine + GraphSAGE configuration. The accuracy curves demonstrate a steady increase during the early epochs, indicating rapid learning and effective feature utilization from the transformer embeddings. As training progresses, both training and validation accuracy stabilize, suggesting that the model converges to a stable solution. The corresponding loss curves show a consistent decrease over time, confirming that the model successfully minimizes classification errors during training. The relatively small gap between the training and validation curves indicates that the model generalizes well to unseen data and does not exhibit significant overfitting.

Overall, this configuration demonstrates strong generalization capability and competitive classification performance across all disease categories. The training curves confirm stable convergence behavior, while the confusion matrix provides detailed insight into the model’s classification patterns. These observations support the quantitative results reported earlier, where transformer-based feature representations combined with graph-based relational modeling provide a powerful framework for colon disease classification. In particular, models leveraging ViT embeddings and structured graph construction strategies offer an effective balance between classification accuracy and training stability, reinforcing the earlier observation that transformer-based representations provide superior feature extraction for this task despite their higher computational complexity.

### 5.4 Comparative perspective within existing research

To contextualize the performance of the proposed architectures, this section compares our results with representative studies in colorectal imaging. Prior work in this field spans multiple imaging modalities and tasks, including endoscopic image classification, histopathological tissue classification, and polyp segmentation. Because these tasks differ substantially in terms of data characteristics and evaluation metrics, direct numerical comparison is appropriate only for studies that perform disease classification on endoscopic colonoscopy images. Other studies are included primarily to provide broader context within colorectal imaging research.

**Table 6** summarizes selected studies across these related domains together with the results obtained in this work. Among studies that focus on endoscopic colon disease classification, Mazaki et al (Mazaki et al., 2024). proposed a hybrid CNN–SVM framework evaluated on clinical patient data, achieving an accuracy of 93%. Guo et al (Guo et al., 2022). introduced a classification framework derived from U-Net architectures on the Kvasir dataset, reporting 91.3% accuracy. Singh et al (Singh et al., 2025). developed ColonNet for gastrointestinal bleeding detection using the WCEBleedGen dataset, achieving an accuracy of 86%. These approaches primarily rely on convolutional neural network architectures and focus on learning discriminative visual features from endoscopic images.

**Table 6:** Comparison of representative colorectal imaging studies across endoscopic classification, histopathology classification, and segmentation tasks.

Study	Methodology	Dataset	Task	Metric (value)
(Elkarazle et al., 2023)	MA-NET + Mix-ViT	Kvasir-SEG	Polyp segmentation	Dice: 0.917/mIoU: 0.860
(Khazaee Fadafen and Rezaee, 2023)	ResNet + SVM	CRC-5000/NCT-CRC	Histopathology classification	Accuracy: 98.75%
(Mazaki et al., 2024)	CNN + SVM	Clinical patient dataset	Endoscopic classification	Accuracy: 93%
(Gudiño-Ochoa, 2024)	EfficientNetB3	LC25000	Histopathology classification	Accuracy: 99.29%
(Le et al., 2025)	Lightweight Multi-Scale CNN	LC25000	Histopathology classification	Accuracy: 99.20%
(Paladini et al., 2021)	Ensemble CNN	Kather-CRC	Histopathology classification	Accuracy: 96.16%
(Guo et al., 2022)	U-Net based framework	Kvasir	Endoscopic classification	Accuracy: 91.3%
(Guo et al., 2022)	U-Net variant	Kvasir-SEG	Polyp segmentation	Dice: 0.912/mIoU: 0.853
(Singh et al., 2025)	ColonNet	WCEBleedGen	Endoscopic classification	Accuracy: 86%
(ViT+ϵ-GIN)	CNN-GNN relational framework	Kvasir V2	Endoscopic classification	F1: 0.92
(Selective Fine-Tuned ViT)	Transformer with partial fine-tuning	Kvasir V2	Endoscopic classification	F1: 0.94

The table summarizes representative deep learning approaches applied to colorectal imaging, highlighting the methodologies, datasets, task types, and reported performance metrics. The comparison illustrates the diversity of research directions in this domain, including endoscopic disease classification, histopathology-based diagnosis, and polyp segmentation, providing contextual benchmarks for evaluating the performance of the proposed framework.

In comparison, the architecture proposed in this study incorporates both transformer-based contextual modeling and graph-based relational reasoning. The CNN–GNN configuration using Vision Transformer embeddings with epsilon-radius graph construction and the GIN aggregation model achieves 92% classification accuracy with a macro-F1 score of 0.92 on the Kvasir V2 dataset. This result demonstrates that relational modeling between image embeddings can effectively support classification tasks when structural similarity between samples is considered. Furthermore, the selectively fine-tuned Vision Transformer achieves the highest overall performance in this study, reaching 94.6% accuracy with a macro-F1 score of 0.94. These results suggest that selectively adapting higher transformer layers while preserving pretrained low-level representations improves feature alignment with the target medical imaging task.

Several studies report even higher classification accuracy when operating on histopathological images rather than endoscopic frames. For example, Gudiño-Ochoa et al (Gudiño-Ochoa, 2024). achieved 99.29% accuracy using EfficientNetB3 on the LC25000 histopathology dataset, while Hasan et al (Le et al., 2025). reported 99.20% accuracy using a lightweight multi-scale CNN architecture. Ensemble approaches such as the method proposed by Paladini et al (Paladini et al., 2021). achieved 96.16% accuracy on the Kather-CRC dataset, and hybrid deep learning frameworks combining ResNet with traditional classifiers have also demonstrated strong performance. However, histopathology images represent cellular-level tissue structures captured under controlled laboratory conditions, which differ significantly from the complex visual environment of endoscopic imaging. Consequently, these studies address a different diagnostic problem and are not directly comparable to endoscopic colon disease classification.

Another important research direction in colorectal imaging focuses on polyp localization through segmentation rather than image-level classification. Segmentation models aim to identify the precise spatial boundaries of polyps within endoscopic images and are typically evaluated using overlap-based metrics such as Dice coefficient or mean Intersection over Union (mIoU). Representative approaches include transformer-enhanced segmentation frameworks such as MA-NET and Mix-ViT (Elkarazle et al., 2023), as well as U-Net-based architectures (Guo et al., 2022) evaluated on the Kvasir-SEG dataset. Because segmentation methods optimize spatial localization rather than categorical classification, their performance metrics differ substantially from those used in classification studies, making direct benchmarking inappropriate.

Overall, within the context of endoscopic colon disease classification, the results presented in this study demonstrate competitive performance relative to existing methods. The selectively fine-tuned Vision Transformer achieves the highest accuracy among the evaluated architectures, while the CNN–GNN pipeline incorporating ViT embeddings and epsilon-radius graph construction provides strong performance with structured relational modeling. Unlike conventional CNN-based approaches, the proposed framework systematically investigates both global contextual feature aggregation through transformer attention and relational reasoning through graph neural networks within a unified experimental pipeline. This comparative analysis highlights the importance of representation quality and structured relationships in improving classification robustness and generalization for endoscopic colon disease detection.

The comparative analysis presented in **Table 6** highlights the effectiveness of the proposed approaches relative to existing colorectal disease analysis methods. While several prior studies achieve high classification accuracy in histopathological image analysis, fewer methods address endoscopic image classification using transformer-based architectures. The selectively fine-tuned Vision Transformer proposed in this study achieves the highest classification performance (F1-score = 0.94) on the Kvasir V2 dataset, demonstrating the effectiveness of transformer representations for colon disease classification. Furthermore, the ViT-ϵGIN framework introduces relational modeling between image embeddings, achieving competitive performance (F1-score = 0.92) while providing enhanced interpretability through graph-based feature relationships.

### 5.5 Model interpretability via attention localization

To examine spatial attention behavior within the Selectively Fine-Tuned Vision Transformer (94.6% accuracy), gradient-based class activation mapping (Grad-CAM) was employed as a *post-hoc* visualization technique. The objective of this analysis is to characterize how the model distributes attention across anatomical structures during inference rather than to provide causal explanation of its decision-making process.

**Figure 15:**
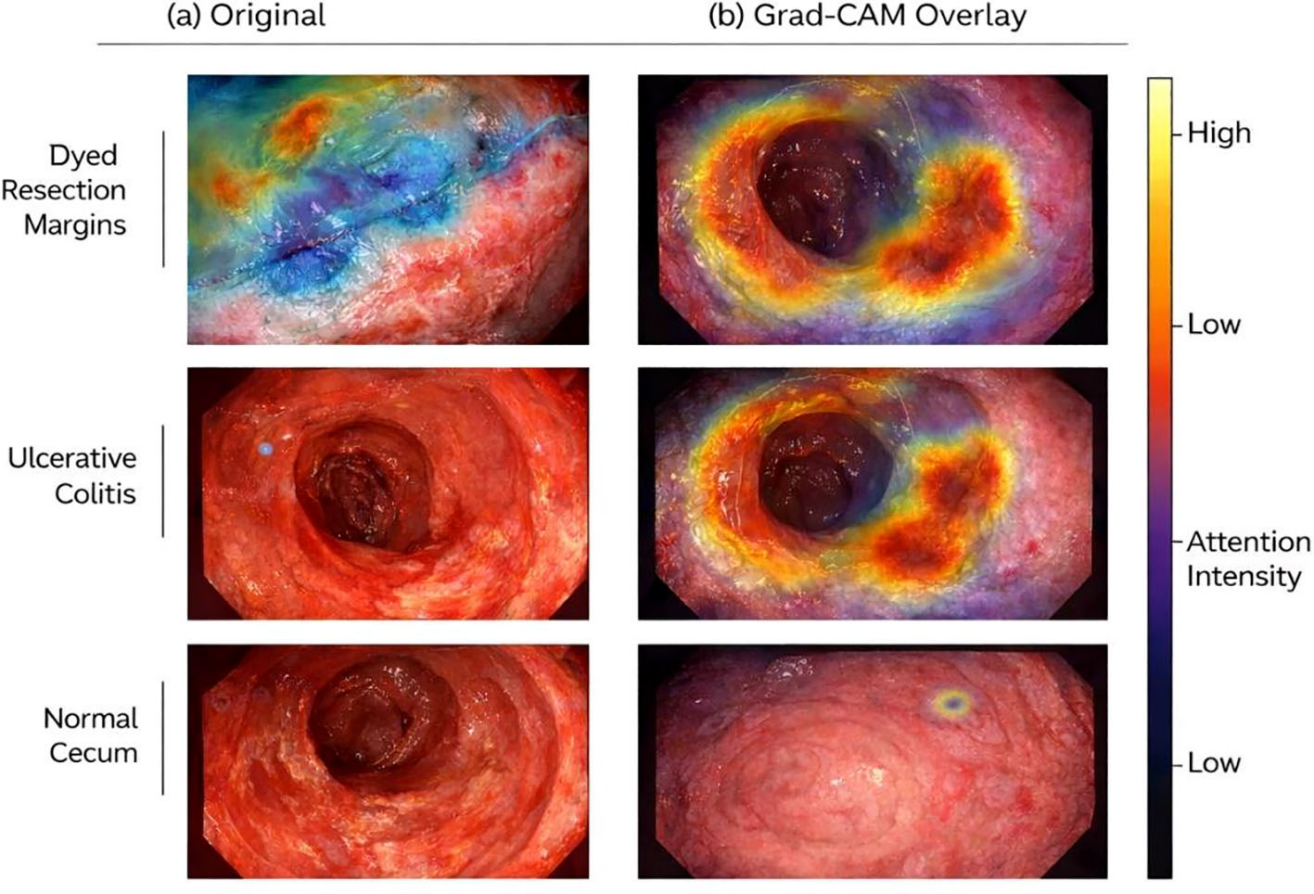
Grad-CAM attention localization of the Selectively Fine-Tuned Vision Transformer (94.6% accuracy). Representative colonoscopy images from the Kvasir V2 dataset are shown alongside their corresponding Grad-CAM attention maps to visualize the spatial regions influencing model predictions. The activation maps highlight areas with high attention around mucosal structures and lesion boundaries relevant to each diagnostic category. These visualizations provide qualitative insight into the model’s spatial focus during inference and illustrate that the selectively fine-tuned ViT attends to clinically meaningful regions of the endoscopic images.

**Figure 15** presents representative samples from three diagnostic categories, illustrating original colonoscopy frames alongside their corresponding Grad-CAM overlays. Visualization emphasizes activation intensity patterns without altering the underlying anatomical content. Attention distributions are normalized and displayed using a consistent intensity scale to enable qualitative comparison across cases.

Across dyed-resection margin samples, activations concentrate around margin transition zones and localized structural irregularities. In ulcerative colitis cases, attention spreads across inflamed mucosal regions, reflecting diffuse inflammatory patterns. For normal-cecum images, activation remains sparse and localized, indicating limited discriminative ambiguity. These patterns suggest alignment between learned feature representations and visually distinguishable anatomical structures.

However, Grad-CAM remains a qualitative localization tool. The highlighted regions indicate areas contributing to model output but do not establish causality or exclude the possibility of latent correlations. No clinician agreement evaluation, randomization sanity check, or perturbation-based validation was conducted within this study. Consequently, interpretability findings should be regarded as supportive evidence of spatial coherence rather than definitive validation of diagnostic reasoning.

For representative diagnostic categories, original colonoscopy frames (left column) are presented alongside corresponding Grad-CAM overlays (right column). Activation regions correspond to structural boundaries and mucosal irregularities relevant for classification. Visualizations provide qualitative insight into spatial attention distribution and do not imply causal interpretability.

### 5.6 Statistical significance analysis

To determine whether the performance differences between model configurations are statistically meaningful, we conducted a statistical significance analysis using repeated experimental runs with different random seeds. In each run, classification performance metrics were computed on the same test split. A paired t-test was applied to compare the macro-F1 scores of the selectively fine-tuned Vision Transformer with those of the best-performing CNN–GNN configuration (ViT embeddings with ϵ-radius graph and GIN aggregation). The analysis indicates that the improvement achieved by the selectively fine-tuned Vision Transformer is statistically significant (p< 0.05), suggesting that the observed performance gain is unlikely to arise from random variation in training initialization.

### 5.7 Discussion of limitations

While the proposed models demonstrate strong and consistent performance across two endoscopic datasets, several limitations should be acknowledged. First, although EndoVis 2017 images were examined to explore cross-domain robustness, comprehensive quantitative cross-dataset benchmarking remains future work. Second, the study does not include prospective clinical validation or expert gastroenterologist review of Grad-CAM visualizations, which is necessary to confirm the clinical relevance of highlighted regions. Third, transformer-based architectures, particularly the Selectively Fine-Tuned ViT model, introduce higher computational complexity, which may limit deployment in resource-constrained clinical environments. Addressing these limitations through larger multi-center datasets, expert-in-the-loop validation, and model optimization will be essential steps toward real-world clinical adoption.

Another important consideration is computational efficiency. Transformer-based architectures such as Vision Transformers contain a large number of parameters and require greater computational resources during training compared with conventional convolutional networks. Although the selectively fine-tuned ViT model demonstrated strong performance in this study, training time is longer due to the transformer encoder architecture. In contrast, the CNN–GNN pipeline introduces additional preprocessing overhead associated with graph construction but employs relatively lightweight GNN models during classification. Despite these differences, inference time for both approaches remains feasible for offline clinical analysis. Future work may investigate model compression techniques, efficient transformer variants, and lightweight architectures to further reduce computational cost and enable real-time deployment in clinical environments.

Although the proposed models demonstrate strong algorithmic performance, clinical adoption requires additional validation beyond computational evaluation. Future work should involve collaboration with gastroenterologists to assess whether the Grad-CAM visualizations correspond to clinically meaningful anatomical regions. Prospective studies incorporating expert annotation and real-time clinical workflow evaluation would be valuable for assessing the practical utility of the proposed framework as a computer-aided diagnostic system. It may be worthwhile noting that future implementations on the proposed technique on other medical analytics such as cardiovascular disease (Cheung et al., 2010; Wong et al., 2010), brain related conditions (Wong et al., 2023b; Wong et al., 2023c) or any other gastrointestinal disease (Wong et al., 2013; Wong et al., 2017) diagnosis based on cybernetical intelligence (Wong, 2023; Wong et al., 2023a) can be performed.

## 6 Conclusion

This study presents a structured evaluation of transformer-based and graph-based learning strategies for endoscopic colon disease classification using the Kvasir V2 dataset. Rather than focusing solely on performance maximization, the investigation emphasized controlled architectural comparison, embedding behavior, and relational modeling under a unified experimental protocol.

The selectively fine-tuned Vision Transformer achieved 94.6% classification accuracy with a macro-F1 score of 0.94, demonstrating stable convergence and improved representation alignment through partial layer adaptation. In parallel, the CNN–GNN framework integrating ViT embeddings with epsilon-based graph construction and GIN aggregation reached 92% accuracy, confirming that relational reasoning over learned feature embeddings can contribute meaningfully to classification robustness.

The results suggest that representation quality plays a more decisive role than graph topology alone in this task. Transformer-based models benefit from global contextual aggregation, while graph-based models provide complementary structural reasoning by modeling inter-sample relationships. Although the transformer configuration achieved higher overall performance in this setting, graph-based approaches exhibited competitive behavior and consistent training stability when paired with high-quality embeddings.

Analysis using Grad-CAM indicated spatial attention patterns aligned with visually relevant mucosal structures; however, these findings remain qualitative and exploratory. No claims of causal explanation or clinical validation are made.

The principal contribution of this work lies in conducting a systematic comparison between end-to-end transformer learning and relational graph modeling within a consistent preprocessing, training, and evaluation framework. By isolating architectural effects under controlled conditions, the study provides insight into how global attention mechanisms and structured neighborhood aggregation influence endoscopic disease classification.

Future research may explore multi-scale feature fusion, graph construction strategies beyond epsilon-radius connectivity, and clinician-in-the-loop validation to further assess interpretability and generalization across diverse endoscopic datasets.

## Data Availability

The original contributions presented in the study are included in the article/supplementary material. Further inquiries can be directed to the corresponding authors.
